# Chemical characterization and metabolic profiling of Xiao-Er-An-Shen Decoction by UPLC-QTOF/MS

**DOI:** 10.3389/fphar.2023.1219866

**Published:** 2023-11-02

**Authors:** Ruipei Yang, Lifang Wei, Jie Wang, Shiying Huang, Pingli Mo, Qiugu Chen, Ping Zheng, Jihang Chen, Shangbin Zhang, Jianping Chen

**Affiliations:** ^1^ Shenzhen Key Laboratory of Hospital Chinese Medicine Preparation, Shenzhen Traditional Chinese Medicine Hospital, The Fourth Clinical Medical College of Guangzhou University of Chinese Medicine, Shenzhen, Guangdong, China; ^2^ School of Medicine, Life and Health Sciences, The Chinese University of Hong Kong, Shenzhen, China; ^3^ KMHD GeneTech Co., Ltd., Shenzhen, Guangdong, China; ^4^ Shenzhen People’s Hospital, Shenzhen, Guangdong, China

**Keywords:** tic disorder, traditional Chinese medicine, Xiao-Er-An-Shen Decoction, chemical characterization, metabolic profiling, UPLC-QTOF/MS

## Abstract

**Background:** Xiao-Er-An-Shen decoction (XEASD), a TCM formula composed of sixteen Chinese medicinal herbs, has been used to alleviate tic disorders (TD) in clinical practice for many years. However, the chemical basis underlying the therapeutic effects of XEASD in the treatment of TD remains unknown.

**Purpose:** The present study aimed to determine the major chemical components of XEASD and its prototype compounds and metabolites in mice biological samples.

**Methods:** The chemical constituents in XEASD were identified using ultra-high Performance liquid chromatography coupled with quadrupole time-of-flight tandem mass spectrometry (UPLC-Q-TOF-MS/MS). Following this, XEASD was orally administered to mice, and samples of plasma, urine, feces, bile, and tissue were collected in order to identify effective compounds for the prevention or treatment of TD.

**Result:** Of the total 184 compounds identified to be discriminated in the XEASD, comprising 44 flavonoids, 26 phenylpropanoids, 16 coumarins, 16 triterpenoids, 14 amino acids, 13 organic acids, 13 alkaloids, 13 ketones, 10 cyclic enol ether terpenes, 7 citrullines, 3 steroids, and 5 anthraquinones, and others. Furthermore, we summarized 54 prototype components and 78 metabolic products of XEASD, measured with biological samples, by estimating metabolic principal components, with four prototype compounds detected in plasma, 58 prototypes discriminated in urine, and 40 prototypes identified in feces. These results indicate that the Oroxylin A glucuronide from Citri reticulatae pericarpium (CRP) is a major compound with potential therapeutic effects identified in brain, while operating positive effect in inhibiting oxidative stress *in vitro*.

**Conclusion:** In summary, our work delineates the chemical basis underlying the complexity of XEASD, providing insights into the therapeutic and metabolic pathways for TD. Various types of chemicals were explored in XEASD, including flavonoids, phenylpropanoids, coumarins, organic acids, triterpenoid saponins, and so on. This study can promote the further pharmacokinetic and pharmacological evaluation of XEASD.

## 1 Introduction

Tic disorder (TD) is a chronic neuropsychiatric condition characterized by motor and vocal tics lasting at least 1 year, including Tourette syndrome (TS) (McGuire et al., 2020). It typically develops in early childhood, with a global prevalence estimated to be between 0.3% and 1%. In China, the prevalence of TD is approximately 6.1% (Scharf et al., 2015). Children with TD often experience co-occurring psychiatric disorders, such as obsessive-compulsive disorder (OCD), attention-deficit/hyperactivity disorder (ADHD), mood and anxiety disorders, and compulsive-like conditions such as hair-pulling and pathological skin-picking (Willsey et al., 2017; McGuire et al., 2021). These conditions, along with pain or injury, social isolation or bullying, and emotional problems, can seriously affect the quality of daily life for TD patients (Brander et al., 2021). Unfortunately, current psychotropic drug therapies have limited effectiveness in treating tics and TD, and may cause significant long-term side effects (Osland et al., 2018; Pringsheim et al., 2019). While first-generation typical antipsychotics like haloperidol have been used in treating TD by antagonizing dopamine receptor type 2 (D2R) in the brain, they are no longer considered first-line drugs due to potential toxicity (Pringsheim et al., 2019). Therefore, it is necessary and urgent to develop new therapeutics for TD.

Traditional Chinese medicine (TCM) has been recognized as an integral part of modern medicine, serving as a vital resource of natural medicines and playing a significant role in the treatment of various human diseases (Li and Kan, 2017). The distinctive characteristic of TCM preparations is the use of multiple herbs, which contain numerous active ingredients that work synergistically on various targets to treat diseases, thereby enhancing the therapeutic effects and minimizing toxicity (Han et al., 2020). Therefore, it is crucial to elucidate the chemical compositions and metabolite profiles of TCM to facilitate their standardization research.

Xiao-Er-An-Shen decoction (XEASD) is a Chinese herbal formulation prepared from sixteen individual herbs, including *Acorus tatarinowii Schott* (Acori Tatarinowii Rhizoma, ATR), *Polygala tenuifolia Willd.* (Polygalae Radix, POR), *Astragalus membranaceus (Fisch.) Bge. var. mongholicus (Bge.) Hsiao* (Astragali Radix, AR), *Arisaema erubescens (Wall.) Schott* (Arisaema Cum Bile, ACB), *Citrus reticulata Blanco* (Citri reticulatae pericarpium, CRP), *Alpinia oxyphylla Miq.* (Alpiniae oxyphyllae Fructus, AOF), *Citrus aurantium L.* (Aurantii Fructus, AF), *Pinellia ternata (Thunb.) Breit.* (Pinelliae Rhizoma, PIR), *Notopterygium incisum Ting ex H. T. Chang* (Notopterygii Rhizoma et Radix, NRR), *Poria cocos (Schw.) Wolf* (Poria, P), *Triticum aestivum L.* (Fructus Tritici Levis, TLF), *Glycyrrhiza uralensis Fisch.* (Glycyrrhizae Radix et Rhizoma, GRR), *Cornus officinalis Sieb. et Zucc.* (Corni Fructus, C), *Hordeum vulgare L.* (Hordei Fructus Germinatus, HFG), *Crataegus pinnatifida Bge. var. major N. E. Br.* (Crataegi Fructus, GF), and Massa Medicata Fermentata (ML) (Supplementary Table S1). It has been widely used in clinics for over 20 years for the treatment of TD (Chen et al., 2019). The efficacy and safety of XEASD have been demonstrated in several studies. In a randomized, double-blind double-dummy clinical research, XEASD was found to be more effective and safer than the haloperidol and aripiprazole groups in improving the number and frequency of muscle movements (Liu et al., 2021).

Our previous *in vivo* studies have demonstrated that the mechanism of action of XEASD in alleviating twitch symptoms and related disorders is mainly related to reversing abnormal changes in neurotransmitter levels and enhancing the antioxidant status of the mouse brain, while *in vitro* experiments have illustrated its ability to modulate neuronal growth and antioxidant activity, thereby providing neuroprotective effects (Li et al., 2018; Chen et al., 2019). Both *in vitro* and *in vivo* results demonstrated the XEASD–induced increase in plasma cyclic adenosine monophosphate (cAMP) levels and the subsequent phosphorylation of cAMP response element-binding (CREB) protein. Although a quality standard has been established to detect the contents of glycyrrhizin, mauroisoflavone glucoside, ammonium glycyrrhizinate, naringin, 3,6′-diglucosyl sucrose, hesperidin and neohesperidin in XEASD, there is still controversy and a lack of understanding regarding the pharmacodynamic material basis of XEASD (Hou-ming. et al., 2018). Hence, a systematic study of the active ingredients and metabolite profiles of XEASD is urgently needed.

Over the last decade, the use of UPLC-QTOF/MS has enabled the rapid and accurate identification of chemical compounds in complex Chinese medicines, natural products and formulas. This has driven the development of natural product analysis and drug design. In the present study, an analytical method of major ingredients based on the UPLC-QTOF/MS system was established. Unknown ingredients were categorized according to the fragmentation patterns and diagnostic ions of different structural types of ingredients. To further characterize XEASD components *in vivo*, the prototypes were analyzed in plasma, urine, feces, and bile, utilizing feature-based similarity in mass spectrometry response and chromatographic retention time. The relationship between bio-transformation and the role of bio-transformed metabolites was identified through mass defect filtration (MDF) and further corroborated by MS/MS spectroscopy.

## 2 Materials and methods

### 2.1 Chemicals and reagents

XEASD granules were obtained from the Pharmaceutical Department of Shenzhen Traditional Chinese Medicine Hospital [Approval number: Guangdong Pharmaceutical Preparation Z20070083], using a combination of the following Chinese medicinal materials [authenticated by Prof. S.B. Zhang (Guangzhou University of Chinese Medicine, Guangzhou, China)] based on the botanical traits recorded in the plant list (http://www.theplantlist.org and http://mpns.kew.org) (Supplementary Figure S1). Neochlorogenic acid, Chlorogenic acid, Cryptochlorogenic acid, Esculetin, Caffeic acid, Loganin, Liquiritin, Nicotiflorin, Ferulic acid, Naringin, Cornuside, Hesperidin, Neohesperidin, Baicilin, Isoliquiritin, Calycosin, Glycyrrhizic acid, Limonin, Nobiletin, Obacunone were obtained from Chengdu Alfa Biotechnology Co., Ltd (Sichuang, China). MTT, palmitic acid and L-glucose (Sigma, USA). Oroxylin A glucuronide [Oroxyloside (purity >98%)], purchased from ACMEC Biochemical Co., Ltd. (Shanghai, China). For the stock solution, Oroxylin A glucuronide was dissolved in 100% dimethyl sulfoxide (DMSO). Bovine serum albumin (BSA) was purchased from Sigma–Aldrich (St. Louis, MO). Control groups received the same volume of solvent DMSO. The purity of each standard compound reported by HPLC analysis was more than 98%. All solutions were prepared from Milli-Q water (Milli-Q Ultrapure water systems, Millipore). Acetonitrile (LC-MS Grade, Optima) and formic acid (LC-MS Grade, Thermo Scientific Pierce) were purchased from Fisher Chemicals.

### 2.2 Mice treatment and sample collection

Male ICR mice (18–20 g, *n* = 8 in each group) were assigned randomly to one of three groups: including a control group for collecting blank bio-samples, a treatment group for collecting plasma, urine, feces, and tissues, and a treatment group for collecting bile. Mice in the control group were administered normal saline (NS) intragastrically. In the treatment group, mice were deprived of food (fasted) for 16 h before the administration of XEASD at the dose of 8 g/kg with free access to drinking water (Chen et al., 2019). Blood samples were collected from the retro-orbital plexus of the mice at 0.25, 0.5, 1, 2, 4, 6, 8, and 10 h after treatment and placed into heparin anticoagulant tubes. The tubes were then centrifuged at 3,000 rpm for 10 min to obtain plasma samples, which were combined for each time point and stored at −80°C until further analysis. Feces, urine, and bile acid samples were collected from each mouse at the indicated time points. This animal study was approved by the Ethics Committees of the Chinese University of Hong Kong (Shenzhen), and was conducted in accordance with the Chinese University of Hong Kong (Shenzhen) animal care regulations (CUHKSZ-AE202206).

### 2.3 Biological sample preparation

Plasma samples were collected and prepared by mixing approximately 200 μL plasma with 600 μL of acetonitrile containing 0.2% methanoic acid. After vortexing for 2 min, the samples were centrifuged at 13,000 rpm, 4°C for 10 min. Then, 400 μL of the supernatant was removed, dried under nitrogen gas, and redissolved in 200 μL of 50% acetonitrile/50% water. Finally, the samples were centrifuged at 13,000 rpm, 4°C for 10 min. An aliquot of 2 μL sample was injected into UPLC-QTOF-MS.

The urine and feces samples were collected every 2 hours by placing mice in individual metabolic cages. The urine was centrifuged at 1,503 *g* (4,000 rpm) for 10 min, after which 1.5 mL of the supernatant was loaded onto a C_18_ solid-phase extraction (SPE) column (Sep-Pak Vac 3 cc 500 mg, Waters, Ireland). The eluant was dried under nitrogen at room temperature and resuspended in 400 μL acetonitrile/water (1:1, v/v) before analysis. Fecal samples that were prepared by weighing approximately 300 mg of feces was placed in 2-mL polypropylene tube, and two volumes of methanol were then added to mix. Fecal extracts were homogenized with 2 mL tungsten carbide beads using a tissue grinder (Wuhan Servicebio, Wuhan, China) and centrifuged for 10 min (13,000 rpm, 4°C), 400 μL of the supernatant were dried. The residue was reconstituted with 200 μL 50% acetonitrile in water (v: v), and the aliquot of 10 μL was injected into the LC–MS/MS system.

Mice were anesthetized with urethane (2.0 g/kg) and bile was collected continually via a cannula inserted into the bile duct at 2, 4, 6, 8, and 10 h, which drained into a collection tube. The bile sample was centrifuged at 4,000 rpm for 10 min, twice. Samples were then loaded on a 1.5 µL C18 pre-column (Optimize Technologies) and the procedure was the same format as urine. For the mice in the three groups, tissue, including liver, heart, spleen, kidney, lung, and brain, were collected after mice execution, respectively. A tissue lyser (Wuhan Servicebio, Wuhan, China) was used to homogenize 100 mg of tissues in 800 μL of methanol. After centrifuging the mixture for 15 min at 4°C at 13,000 rpm, 400 μL of the supernatant were dried under nitrogen gas. The residue was reconstituted with 100 μL 50% acetonitrile in water (v: v), and the aliquot of 10 μL was injected into the LC–MS/MS system.

### 2.4 UPLC-QTOF-MS analysis condition

LC&MS/MS experiments were performed on an exion LC system (AB Sciex, Foster City, CA, USA). An Acquity HSS T3 column (1.8 μm, 2.1 × 150 mm) equipped with a VanGuard precolumn (1.8 μm; Waters Corporation) served for chromatographic separation. The mobile phases used for elution were (A) 0.1% (v/v) formic acid/water and (B) acetonitrile. The UPLC eluting conditions were optimized as follows: 3%–7% acetonitrile for 0–5 min, 7%–30% acetonitrile for 5–12 min, 30%–80% acetonitrile for 12–20 min, 80%–95% acetonitrile for 20–21 min, and 95% acetonitrile for 21–27 min, then back to the initial ratio of 3% B and maintained with additional 10 min for re-equilibration. The sample injection volume was 2 μL. MS data were recorded using an AB Sciex X500B QTOF mass spectrometer with an ESI source and operated in both the positive and the negative modes. MS conditions were set as follows: ions spray voltage – 4500 V in negative mode and 5,500 V in positive, ion source heater temperature 500°C, source gas 145 psi, source gas 245 psi, and curtain gas 35 psi. The declustering potential, collision energy and the collision energy spread (CES) were set at 50V, ±35V and 15V, respectively. The initial data was processed on the Sciex OS 1.6.1 platform, followed by metabolite fishing using MetabolitePilot ™ 2.0.4 software (Peak finding strategy combined mass defect filter (MDF), characteristic product ion filter (PIF), and neutral loss filter (NLF). Set MS *m/z* tolerance at 10 ppm and minimum peak intensity at 1,000 cps. Sample-control ratio was set at 3).

### 2.5 Neonatal rat cardiomyocyte culture and treatment

H9C2 (rat cardiomyoblasts; American Type Culture Collection, Manassas, VA) were cultured in Dulbecco’s modified Eagle’s medium (DMEM) with 10% fetal bovine serum and 5% CO_2_ at 37°C. After culturing in DMEM with 10% FBS for 48 h, the cells were pre-treated with Oroxylin A glucuronide (0, 10, 100 μM) for 1 h, followed by Palmitic acid (PA, 0.5 mM) and high glucose (HG, 6 g/L) stimulation for 12 h.

### 2.6 Cell survival and proliferation assays

Cells were evaluated with the 3-(4,5-dimethylthiazol-2-yl)-2,5-diphenyltetrazolium bromide (MTT) assay (Dong et al., 2014; Xu et al., 2018). In a nutshell, 20 μL of MTT solution was added to the culture medium at a final concentration of 0.5 mg mL^−1^ and incubated at 37°C for 4 h. Then, the supernatants were aspirated carefully, 150 μL of DMSO was added to each well to dissolve the reaction product insoluble formazan of MTT, and the OD was spectrophotometrically measured using a microplate reader (BioTek, uQuant, Santa Barbara, CA, USA) at a wavelength of 570 nm, with DMSO as a blank.

### 2.7 Statistical analysis

Statistical analysis was conducted using GraphPad Prism 9 statistical software. Mean values were compared between control and treatment groups using One-way ANOVA analysis. A *p*-value less than 0.05 was considered statistically significant (*), while a *p*-value less than 0.01 was considered highly significant (**).

## 3 Results

### 3.1 Characterization of chemical compounds in XEASD

**FIGURE 1:**
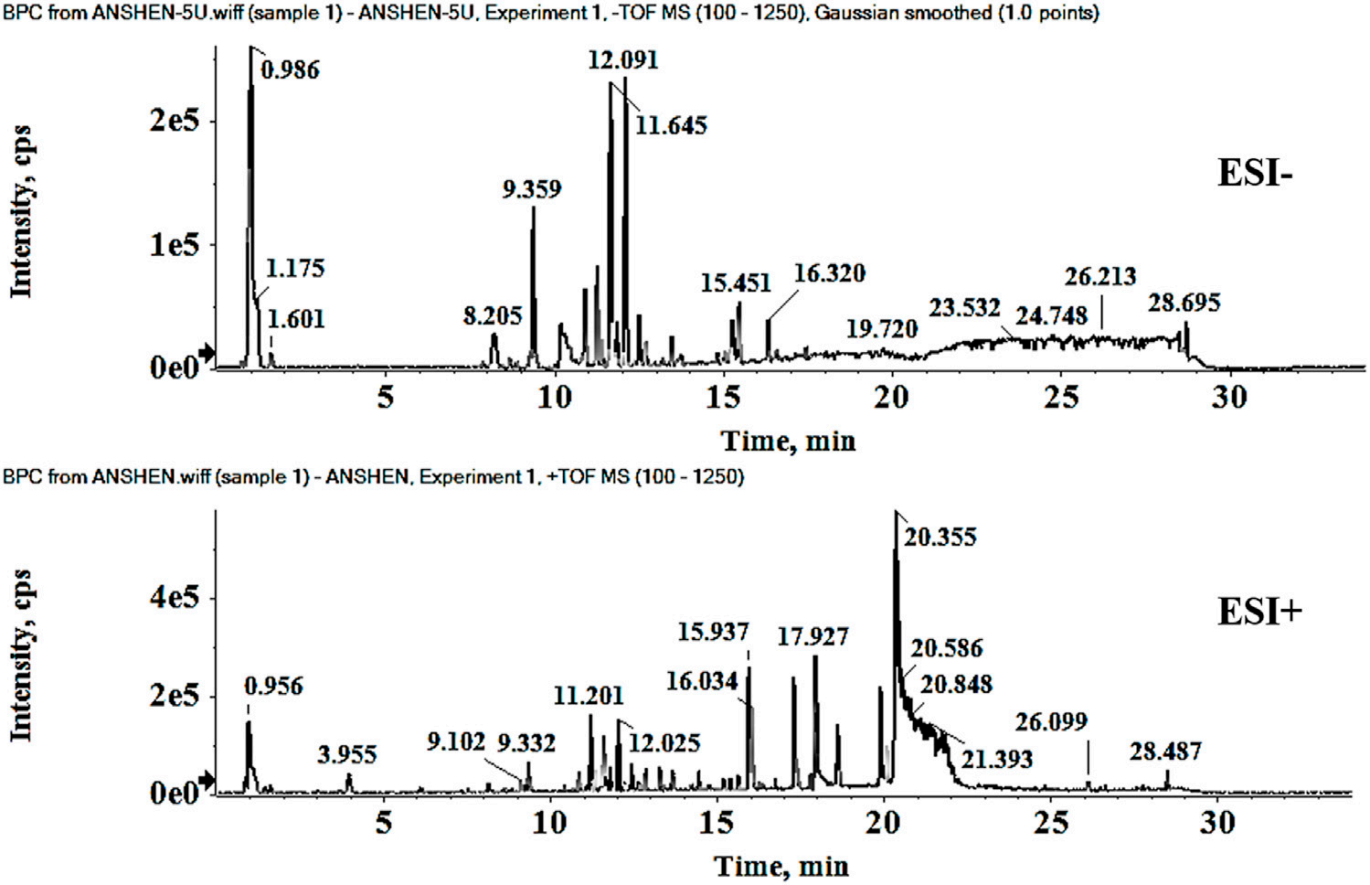
Base peak chromatogram (BPC) of XEASD.

**FIGURE 2:**
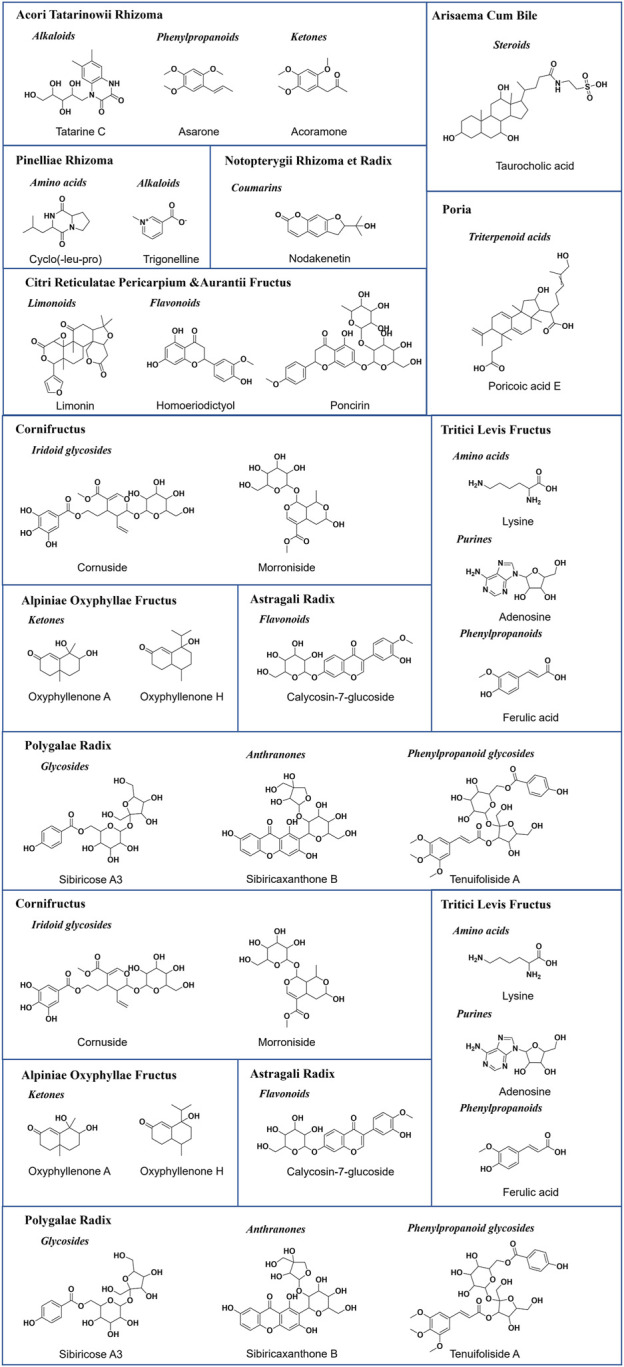
Representative structures of each medicine of XEASD.

Representative base peak chromatogram (BPC) of XEASD in the positive ion mode and negative ion mode are shown in Figure 1. Initial analysis identified or tentatively characterized a total of 198 chemical components by UPLC-QTOF-MS (Supplementary Table S2), including 44 flavonoids, 26 phenylpropanoids, 16 coumarins, 16 triterpenoids, 14 amino acids, 13 organic acids, 13 alkaloids, 13 ketones, 10 cyclic enol ether terpenes, 7 citrullines, 3 steroids, and 5 anthraquinones. Pooled outcome findings are summarized in Table 1. ATR calamus was found to be rich in chemical components, including flavonoids, alkaloids, phenylpropanoids, and ketones, with specific characteristic components such as Acoramone and Tatarine C. ACB cholonan was characterized by characteristic bile acid compounds, while triterpenoids were the characteristic components of P. The major characteristic ingredients of CRP were flavonoid. Coumarins were identified in NRR. C contained cyclic enol ether terpenoids such as loganin and morroniside, while AOF had characteristic naphthones. POR contained anthraquinones and phenylpropanoid glycosides as its characteristic components. AR mainly contained flavonoids in this experiment, while CRP had a high degree of similarity in composition with AF, with a large number of flavonoids, as well as a few coumarins and citrulline. PIR and TLF were mainly composed of amino acid components, while HFG contained Hordenine as its characteristic component, in addition to some organic acids and amino acid compounds. ML was difficult to attribute as a curative component, and only 7 compounds were identified in this experiment, but it contained alkaloids, flavonoids, organic acids, phenylpropanoids, and other components. Finally, GF was mainly characterized by organic acids and phenylpropanoids. Figure 2 illustrates the representative structures of each herb.

**TABLE 1:** Chemical component of XEASD.

	Flavonoids	Phenylpropanoid glycosides	Triterpenoid saponins	Coumarins	Amino acid	Organic acids	Alkaloids	Ketones	Iridoid glycosides	Citrullines	Steroids	Saccharides	Anthranones	Others (Nucleosides, glycosides, esters, etc.)	Total
ATR	5	8	1	2	-	-	4	3	-	-	-	-	1	3	27
ACB	4	7	-	-	1	4	1	-	-	-	3	-	-	1	21
PIR	1	2	-	-	13	-	2	-	-	-	-	-	-	3	21
P	-	-	3	-	-	-	-	-	-	-	-	-	-	-	3
CRP	25	-	-	6	-	-	3	-	-	7	-	-	-	2	43
NRR	-	7	-	11	-	2	-	-	-	-	-	-	-	-	20
C	-	-	-	-	-	3	-	-	10	-	-	-	-	2	15
AOF	-	-	-	-	-	-	-	10	-	-	-	-	-	-	10
POR	-	14	2	-	-	-	-	-	-	-	-	-	4	2	22
AR	5	4	-	-	-	-	-	-	-	-	-	-	-	-	9
AF	21	4	-	5	-	-	-	-	-	4	-	-	-	1	35
TLF	-	2	-	-	12	1	-	-	-	-	-	-	-	2	17
HFG	2	1	-	-	2	3	2	-	-	-	-	-	-	1	11
ML	1	2	-	-	-	1	2	-	-	-	-	-	-	1	7
GF	2	5	2	1	-	4	-	-	1	-	-	-	-	2	17
GRR	14	-	8	-	-	-	-	-	-	-	-	1	-	1	24
Total	44	26	16	16	14	13	13	13	10	7	3	5	5	13	198

The number in the brackets was the repeat compounds.

### 3.2 Fragmentation mechanisms of medicine representative structures

#### 3.2.1 Acori tatarinowii Rhizoma-derived compounds

**FIGURE 3:**
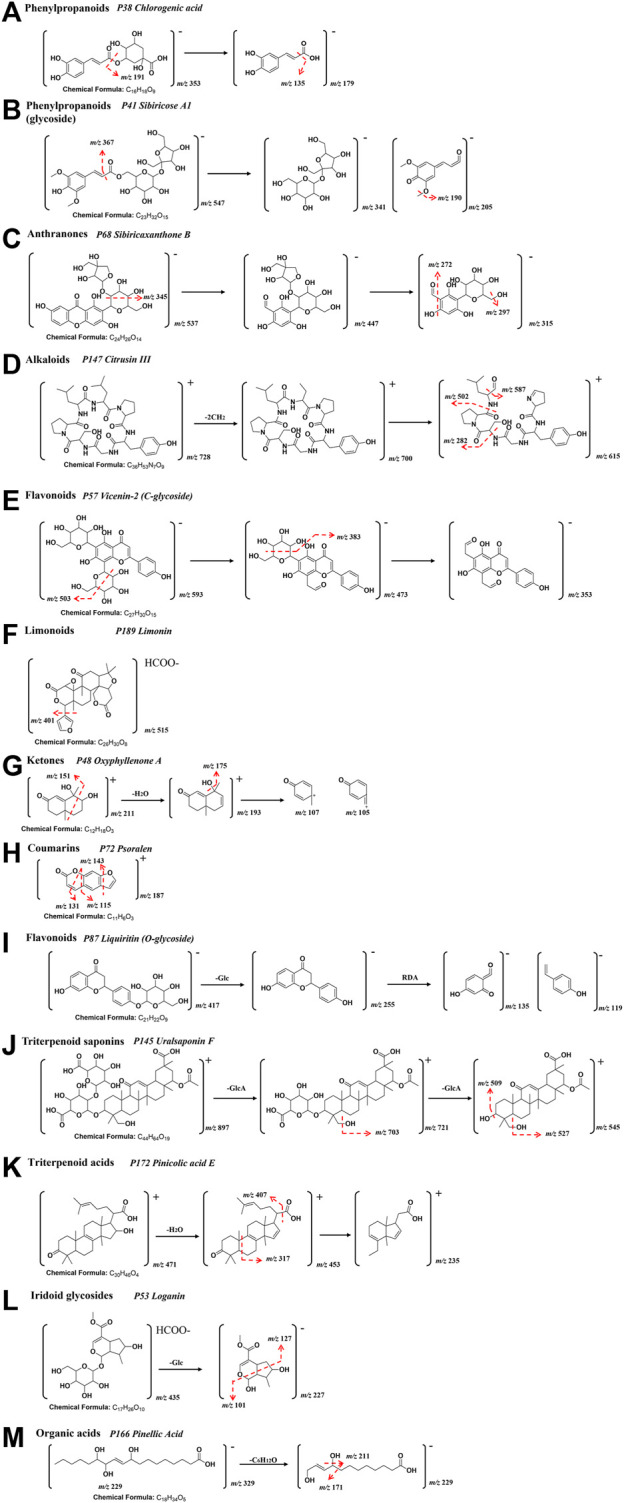
A representative *MS*/*MS* fragmentation spectrums and major fragmentation pattern of XEASD. **(A) P38** Chlorogenic acid*; **(B) P41** Sibiricose A1; **(C) P68** Sibiricaxanthone B; **(D) P147** Citrusin III; **(E) P57** Vicenin-2/Saponarin; **(F) P189** Limonin*; **(G) P48** Oxyphyllenone A; **(H) P72** Psoralen; **(I) P87** Liquiritin*; **(J) P145** Uralsaponin F; **(K) P172** Pinicolic acid E; **(L) P53** Loganin*; **(M) P166** Pinellic Acid.

Identification of compounds in ATR resulted in the detection of 27 components. Among them, phenylpropanoids (**P94** 2,4,5-trimethoxybenzoic acid, **P118** Tatarinoids B, **P126** Alpha-asarone, **P188** Propioveratrone), and alkaloids (**P55** Tatarine C, **P132** N-trans-feruloyl-tyramine, **P183** N-lauryldiethanolamine, **P187** Phytosphingosine) were identified as characteristic components. **P38** Chlorogenic acid* showed [M-H]^−^ in the negative ion at *m/z* of 353.0877 and had fragment ions at *m/z* of 191, which corresponds to [M-C_9_H_8_O_3_-H]^−^. The typical fragmentation pattern of **P38** is drawn in Figure 3A [* indicates that the compound was verified by the control (Supplementary Table S2)].

#### 3.2.2 Polygalae Radix -derived compounds

Twenty-two compounds were detected and identified from Polygalae Radix and 14 of the compounds were phenylpropanoid glycosides(**P36** Sibiricose A5, **P39** Sibiricose A5 isomer, **P41** Sibiricose A1, **P47** Sibiricose A1 isomer, **P58** Sibiricose A6, **P70** Glomeratose A, **P86** Tenuifoliside B,P104 1-O-Sinapoyl-beta-D-glucose, **P105** 3′,6-Disinapoylsucrose, **P109** Polygenin A, **P110** Tenuifoliside A isomer, **P116** Tenuifoliside A, **P127** Tenuifoliside C, **P129** Tenuifoliose J), and 4 of the compounds were Anthranones(**P68** Sibiricaxanthone B, **P73** Polygalaxanthone III, **P74** Irisxanthone, **P134** Polygalaxanthone IV). For identification of sibiricose A1 (**P41**), a phenylpropanoid glycoside, the successive loss of hexose (−162 Da) and H_2_O (−18 Da) was observed in the fragmentation pathway. The compound showed [M-H]^−^ at *m/z* of 547.1668 and [M+H]^+^ at *m/z* of 549.1814. The fragmentation pathways for **P41** were similar to those of **P38**, which are illustrated in Figure 3B. Simple carboxylic acids were typically detected in negative mode, and neutral loss of CH_3_ (15 Da), H_2_O (−18 Da), and CO_2_ (−44 Da) were the most common fragments. **P68** Sibiricaxanthone B exhibited [M-H]^−^ ion at *m/z* of 537.1269 and [M + H]^+^ ion at *m/z* of 539.1399, with a characteristic fragment ion at [M- C_6_H_6_O-H]^−^at *m/z* of 447, [M-C_6_H_6_O-C_5_H_9_O_4_-H]^−^ at *m/z* of 315, [M-C_6_H_6_O-C_5_H_9_O_4_-H_2_O-H]^-^ at *m/z* of 297, [M-C_6_H_6_O-C_5_H_9_O_4_-CH_2_O-H]^−^ at *m/z* of 285, and [M-C_6_H_6_O-C_5_H_9_O_4_-CH_2_O-H_2_O-H]^-^ at *m/z* of 272. The typical fragmentation pathways of **P68** Sibiricaxanthone B are shown in Figure 3C.

#### 3.2.3 Astragali Radix -derived compounds

In total, nine features were identified in Astragali Radix. Among them, phenylpropanoids (**P29** Neochlorogenic acid*, **P38** Chlorogenic acid*, **P42** Cryptochlorogenic acid*, **P102** Isochlorogenic acid A) and flavonoids (**P82** Calycosin-7-glucoside, **P122** Ononin, **P133** 9,10-dimethoxypterocarpan-3-O-β-D-glycoside, **P146** Calycosin*, **P179** Formononetin) were characterized.

#### 3.2.4 Arisaema Cum Bile -derived compounds

Twenty-one compounds were structurally characterized from Arisaema Cum Bileand, including 7 phenylpropanoids (**P29** Neochlorogenic acid*, **P38** Chlorogenic acid*, **P42** Cryptochlorogenic acid*, **P66** 3-O-feruloylquinic acid, **P69** 5-Hydroxyferulic acid, **P91** Ferulic acid*, **P92** Sinapic acid), 3 organic acids (**P16** Succinic acid, **P138** Sebacic acid, **P197** (s)-3-hydroxypalmitic acid), 4 flavonoids (**P67** Isoschaftoside, **P89** Nicotiflorin*, **P107** Hesperidin*, **P111** Neohesperidin*), 3 steroids (**P155** Taurocholic acid, **P159** Glycocholic acid, **P177** 7-O-glycochenodeoxycholic acid), amino acids (**P28** Tryptophan), alkaloids (**P31** Trigonelline), phenol derivatives (**P32** 3,4-dihydroxybenzaldehyde).

#### 3.2.5 Citri reticulatae pericarpium -derived compounds

In total, 43 features were identified in Citri reticulatae pericarpium, with 25 of them being flavonoids (including **P57** Vicenin-2/Saponarin, **P59** Diosmetin-6,8-di-c-glucoside, **P60** Chrysoeriol-6,8-di-c-glucoside, **P64** Naringin 4′-Glucoside, **P78** Neoeriocitrin, **P80** Rutin, **P85** Eriocitrin, **P99** Isorhoifolin, **P101** Rhoifolin, **P103** Narirutin, **P107** Hesperidin*, **P111** Neohesperidin*, **P115** Hesperetin 7-O-Glucoside, **P117** Nomilinic acid glycoside, **P120** Baicilin*, **P135** Oroxylin A Glucoronide, **P141** Poncirin, **P151** Natsudaidain 3-(4-O-3-Hydroxy-3-Methylglutaroylglucoside), P162 Homoeriodictyol, **P168** Monohydroxy-tetramethoxyflavone, **P169** Tangeretin, **P171** Deacetylnomilinic acid, **P175** 7-hydroxy-5,6,8,3′,4′-pentamethoxyflavone, **P184** 3,5,7,4′-tetramethoxyflavone, **P191** Nobiletin*, **P193** 3,5,6,7,8,3′,4′-heptamethoxyflavone), and 3 Alkaloids (**P10** Piperidineacetic acid, **P147** Citrusin III, **P164** Citrusin I). Citrusin III (**P147**, [M+H]^+^at *m/z* of 728.3974) was a representative compound, which produced fragment ions at [M-2CH_2_+H]^+^ at *m/z* of 700, [M-2CH_2_-C_4_H_7_NO +H]^+^ at *m/z* of 615, [M-2CH_2_-C_4_H_7_NO-CHO +H]^+^ at *m/z* of 587, [M-2CH_2_-C_4_H_7_NO- C_6_H_12_NO + H]^+^ at *m/z* of 502, and [M-2CH_2_-C_4_H_7_NO- C_16_H_2_ON_4_O_4_ + H]^+^ at *m/z* of 282. The fragmentation pathways were depicted in Figure 3D. Flavonoids **P57** Vicenin-2/Saponarin displayed an [M-H]^-^ ion at *m/z* of 593.1535 and [M + H]^+^ ion at *m/z* of 595.1643, while it showed characteristic fragment ion at [M- C_3_H_8_O_3_-H]^−^ at *m/z* of 503, [M-C_4_H_10_O_4_-H] ^−^ at *m/z* of 473, [M-C_4_H_10_O_4_-C_3_H_8_O_3_-H] ^−^ at *m/z* of 383, and [M-2C_4_H_10_O_4_-H]^−^ at *m/z* of 353. The typical fragmentation pathways of **P57** Vicenin-2/Saponarin are depicted in Figure 3E. **P189** codonopsine exhibited [M+FA-H]^−^ ion at *m/z* of 515.1944, while the fragment ions at *m/z* of 469 and 401, corresponding to [M-FA-H]^−^ and [M-FA-C_4_H_4_O-H]^−^, respectively. Figure 3F shows the representative fragmentation pathways of **P189** codonopsine.

#### 3.2.6 Alpiniae oxyphyllae fructus -derived compounds

Ten compounds were identified in Alpiniae oxyphyllae Fructus, which include ketones (**P48** Oxyphyllenone A, **P77** Oxyphyllenone B, **P95** Oxyphyllenodiol A, **P100** (11S)-nootkatone-11,12-diol, **P123** Oxyphyllenodiol B, **P124** Teuhetenone A, **P153** 11-hydroxy-valenc-1(10)-en-2-one, **P167** Dehydro-nootkatone, **P186** Oxyphyllenone H, **P190** Oxyphyllone E). Ketone compounds typically undergo neutral loss of H_2_O during fragmentation. For instance, **P48** Oxyphyllenone A had an [M + H]^+^ ion at *m/z* of 211.1327. Its characteristic product ions at *m/z* of 193 [M-H_2_O+H]^+^, 175 [M-2H_2_O+H]^+^, 151 [M-C_3_H_6_O^−^+H]^+^, 107 [M-C_7_H_7_O]^+^, and 105 [M-C_7_H_5_O]^+^were identified. Typical MS/MS fragmentation patterns of **P48** Oxyphyllenone A are illustrated in Figure 3G.

#### 3.2.7 Aurantii Fructus -derived compounds

In this study, flavonoids (**P57** Vicenin-2/Saponarin, **P64** Naringin 4′-Glucoside, **P78** Neoeriocitrin, **P80** Rutin, **P85** Eriocitrin, **P97** Naringin*, **P99** Isorhoifolin, **P101** Rhoifolin, **P103** Narirutin, **P107** Hesperidin*, **P108** Naringenin-7-O-glucoside, **P111** Neohesperidin*, **P141** Poncirin, **P151** Natsudaidain 3-(4-O-3-Hydroxy-3-Methylglutaroylglucoside, **P157** Naringenin, **P162** Homoeriodictyol, **P168** Monohydroxy-tetramethoxyflavone, **P169** Tangeretin, **P184** 3,5,7,4′-tetramethoxyflavone, **P191** Nobiletin*, **P193** 3,5,6,7,8,3′,4′-heptamethoxyflavone), phenylpropanoids (**P29** Neochlorogenic acid*, **P38** Chlorogenic acid*, **P42** Cryptochlorogenic acid*, **P49** Caffeic acid*), coumarins (**P44** Cnidioside A, **P71** p-coumaric acid, **P83** Umbelliferone, **P128** Oxypeucedanin, **P140** Oxypeucedanin hydrate), limonoids (**P84** Limonin glucoside, **P189** Limonin*, **P192** Nomilin, **P194** Obacunone*), and furans (**P25** Phenylalanine) were identified as the characteristic components of AF. The composition of AF and CRP were found to be highly similar, with both containing a large number of flavonoids and a small amount of coumarin and citrulline.

#### 3.2.8 Pinelliae Rhizoma -derived compounds

In total, 21 features could be attributed to Pinelliae Rhizoma, including 13 Amino acids (**P1** Ornithine, **P2** Lysine, **P3** Histidine, **P4** Arginine, **P5** Asparaginic acid, **P6** Threonine, **P7** Glutamic acid, **P8** Serine, **P9** Proline, **P17** Leucine, **P18** Isoleucine, **P25** Phenylalanine, **P61** Cyclo(-leu-pro)), 2 Alkaloids (**P13** Nicotinamide, **P31** Trigonelline), 2 phenylpropanoids (**P91** Ferulic acid*, **P196** Dibutyl phthalate), 2 Purines (**P19** Adenosine, **P21** Guanosine), 1 Phenol derivatives (**P196** Dibutyl phthalate), and 1 Flavonoids (**P67** Isoschaftoside).

#### 3.2.9 Notopterygii Rhizoma et radix -derived compounds

The characteristic compounds in Notopterygii Rhizoma et Radix were identified as coumarins (**P50** Fraxin, **P75** Decuroside V, **P83** Umbelliferone, **P88** Scopoletin, **P93** Nodakenetin, **P96** Marmesinin, **P125** 5-Isopentenyloxy-7-methoxycoumarins, **P131** Marmesin, **P142** 3, 4, 5-trimethoxy-trans-cinnamic acid, **P143** 6-O-trans-feruloyl nodakenin, **P185** p-hydroxyphenethyl ferulate and **P198** Oleic acid) and phenylpropanoids (**P29** Neochlorogenic acid*, **P38** Chlorogenic acid*, **P42** Cryptochlorogenic acid*, **P65** Coumaroyl-glucose). Coumarins were generally detected in the positive ionization mode. Psoralen (**P72**, [M + H]^+^ at *m/z* of 187.0386) showed fragment ions at [M-C_2_H_4_O+ H]^+^ at *m/z* of 143, [M-C_2_H_4_O_2_ + H]^+^ at *m/z* of 131, and [M-C_3_H_4_O_2_+H]^+^ at *m/z* of 115. Typical MS/MS fragmentation patterns of **P72** Psoralen are illustrated in Figure 3H.

#### 3.2.10 Glycyrrhizae Radix et rhizoma-derived compounds

In total, 24 features were identified in Glycyrrhizae Radix et Rhizoma, including 14 flavonoids (**P51** Puerarin, **P67** Isoschaftoside, **P76** Isoviolanthin, **P79** Licoagroside A, **P81** Liquiritin apioside, **P87** Liquiritin*, **P108** Naringenin-7-O-glucoside, **P114** Isoliquiritin Apioside, **P121** Isoliquiritin*, **P122** Ononin, **P136** Liquiritigenin, **P157** Naringenin, **P173** Isoliquiritigenin, **P179** Formononetin), 8 triterpenoid saponins (**P145** Uralsaponin F, **P148** 22-hydroxy-licoricesaponin G2, **P149** Licoricesaponin A3, **P152** 22β-acetoxylglycyrr-hizicacid, **P154** Licoricesaponine G2 isomer, **P165** Licoricesaponine G2, **P174** Glycyrrhizic acid*, **P182** Licoricesaponine H2), one saccharide (**P11** Gentiobiose) and one purine (**P14** Adenine). The representative compound liquiritin* (**P87**, [M-H]^−^ at *m/z* of 417.1194, [M+H]^+^ at *m/z* of 419.1337) showed fragments ions at [M-GlcA-H]^−^ at *m/z* of 255, *m/z* 135 and *m/z* 119 were produced after RDA cracking. Typical MS/MS fragmentation patterns in Figure 3I. Uralsaponin F (**P145**, [M+H]^+^ at *m/z* of 897.4109), showed fragments ions at [M-GlcA-H]^+^ at *m/z* of 721, [M-GlcA-H_2_O+H]^+^ at *m/z* of 703, [M-2GlcA+H]^+^ at *m/z* of 545, [M-2GlcA-H_2_O+H]^+^ at *m/z* of 527, and [M-2GlcA-2H_2_O+H]^+^ at *m/z* of 507.

#### 3.2.11 Poria -derived compounds

The characteristic compounds in Poria Cocos were identified as triterpenoid acids (**P158** Poricoic acid E, **P163** 3-oxo-16α-hydroxylanosta-7,9 (11),24-trien-21-oic acid, **P172** Pinicolic acid E). Triterpenoid acids generally respond in the positive mode. Pinicolic acid E (**P172**, [M + H]^+^ at *m/z* of 471.3467) exhibited fragment ions at [M-H_2_O+ H]^+^ at *m/z* of 453, [M-CH_2_O_2_+H]^+^ at *m/z* of 407, [M- C_9_H_14_O_2_ + H]^+^ at *m/z* of 317, and [M-H_2_O-C_6_H_12_-C_9_H_16_O+H]^+^ at *m/z* of 235. The typical fragmentation pathways of **P172** Pinicolic acid E are shown in Figure 3K.

#### 3.2.12 Fructus Tritici Levis -derived compounds

In Fructus Tritici Levis, 12 compounds were identified, including 7 amino acids (**P2** Lysine, **P3** Histidine, **P4** Arginine, **P5** Asparaginic acid, **P6** Threonine, **P7** Glutamic acid, **P8** Serine, **P9** Proline, **P17** Leucine, **P1**8 Isoleucine, **P25** Phenylalanine, **P28** Tryptophan),2 phenylpropanoids (**P69** 5-Hydroxyferulic acid, **P91** Ferulic acid*), 2 purines(**P19** Adenosine, **P21** Guanosine) and 1 organic acids (**P112** Azelaic acid).

#### 3.2.13 Cornus -derived compounds

In total, 15 features were identified in Cornus, including 10 iridoid glycosides (**P33** Cornusglucosides C, **P34** Loganic acid, **P35** Morroniside, **P46** Polygalatenoside A, **P53** Loganin*, **P56** Sweroside, **P62** Logmalicid A, **P63** Logmalicid B, **P106** Cornuside*, **P137** Cornusfuroside D), three organic acids (**P12** Malic acid, **P15** Citric acid, **P2**3 Gallic acid), one phenol derivatives (**P24** 7-O-galloyl-D-sedoheptulose), and one furan (**P26** 5-HMF). **P53** Loganin* showed the [M+FA-H] ^−^ ion at *m/z* of 435.1518 and [M+H]^+^ ion at *m/z* of 391.1593, while it had characteristic fragment ion at [M-Glc-H] ^−^ at *m/z* of 227, [M-Glc-C_5_H_12_O_2_-H] ^−^ at *m/z* of 127, and [M-Glc- C_6_H_9_O_3_-H]^+^ at *m/z* of 110. Typical MS/MS fragmentation patterns of **P53** are illustrated in Figure 3L.

#### 3.2.14 Hordei Fructus Germinatus -derived compounds

In total, eleven features could be attributed to Hordei Fructus Germinatus, including three organic acids (**P156** 9,10-Dihydroxy-8-oxo-12-octadecenoic acid, **P166** Pinellic Acid, **P195** 9,10-dihydroxy-12Z-octadecenoic acid), two amino acids (**P25** Phenylalanine, **P28** Tryptophan), two flavonoids (**P37** Vitexin, **P57** Vicenin-2/Saponarin), two alkaloids (**P20** N-Methyltyramine, **P22** Hordenine), one phenylpropanoid (**P38** Chlorogenic acid *) and one purine (**P19** Adenosine). **P166** Pinellic Acid exhibited the [M-H]^−^ ion at *m/z* of 329.2343, along with a characteristic fragment ion at [M-C_6_H_12_O-H] ^−^ with *m/z* of 229, [M-C_6_H_12_O-H_2_O-H]^−^ with *m/z* of 211, [M-C_8_H_16_O_2_-H_2_O-H] with *m/z* of 171, and [M-C_8_H_16_O_2_-3H_2_O-H]^+^ with *m/z* of 139. Typical MS/MS fragmentation patterns of **P166** are illustrated in Figure 3M.

#### 3.2.15 Massa Medicata Fermentata-derived compounds

In the current study, Massa Medicata Fermentata was found to contain two alkaloids (**P43** Tetrahydroharman-3-carboxylic acid, **P98** Perlolyrine), two phenylpropanoids (**P69** 5-Hydroxyferulic acid, **P91** Ferulic acid*), one flavonoid (**P107** Hesperidin*), one organic acid (**P166** Pinellic Acid), and one purine (**P19** Adenosine).

#### 3.2.16 Crataegi Fructus -derived compounds

In total, 23 features were identified in Crataegi Fructus, including five phenylpropanoids (**P29** Neochlorogenic acid*, **P38** Chlorogenic acid*, **P42** Cryptochlorogenic acid*, **P69** 5-Hydroxyferulic acid, **P91** Ferulic acid*), four organic acids(**P12** Malic acid, **P15** Citric acid, **P23** Gallic acid, **P40** 2-benzyl-2,3-dihydroxybutanedioic acid), two flavonoids (**P80** Rutin, **P157** Naringenin), one triterpenoid acid (**P161** Euscaphic acid B), one iridoid glycoside (**P34** Loganic acid), and one coumarin (**P71** p-coumaric acid).

### 3.3 Characterization of XEASD-Related xenobiotics in mice biological samples

**FIGURE 4:**
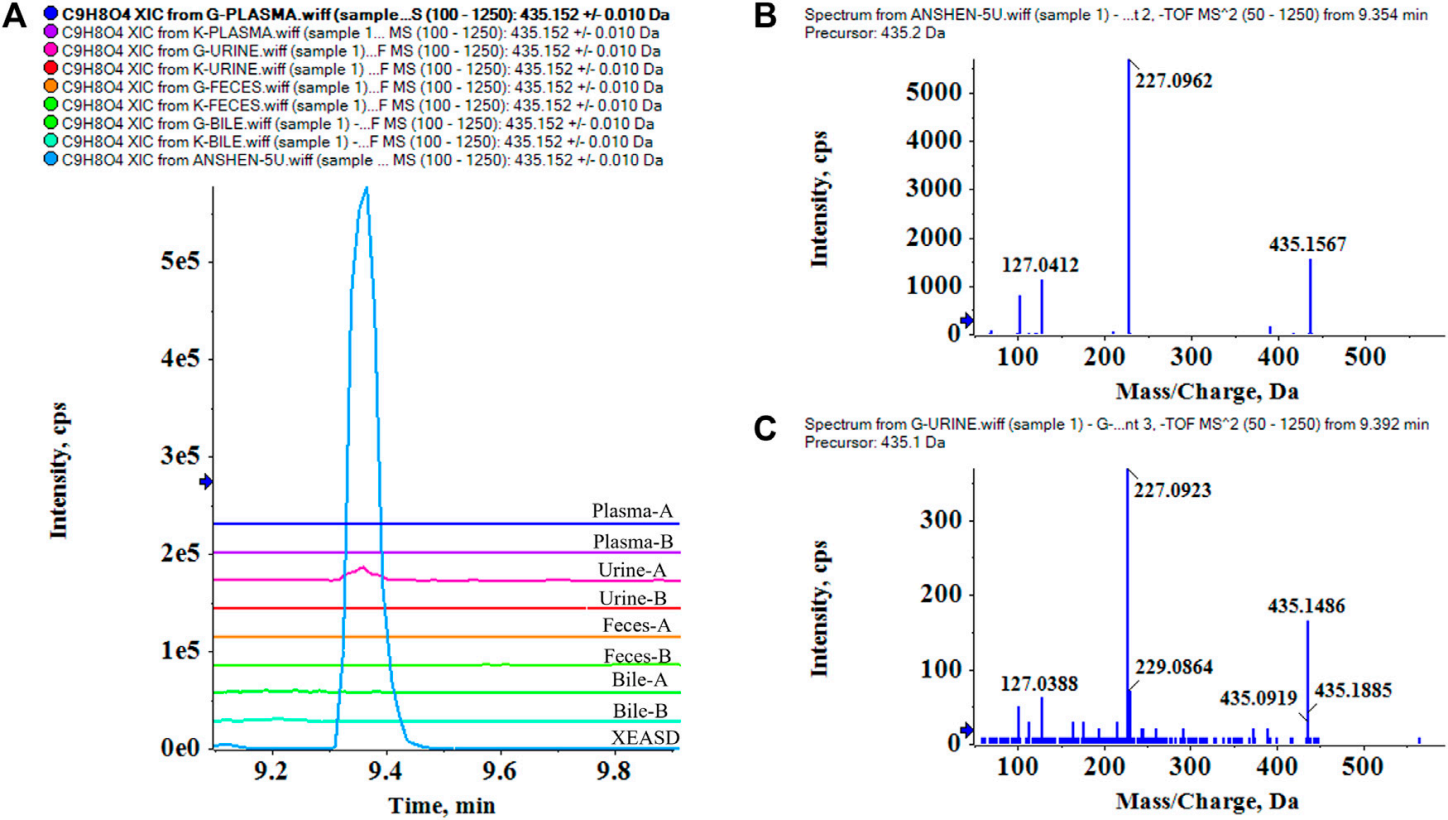
Identification of prototypes in bio-samples, and P53 Loganin is taken as an example. **(A)** multiple XICs of Loganin in XEASD and bio-samples, A- Administration,B-Blank.; **(B)** *MS/MS* spectrum of Loganin in XEASD; **(C)** MS/MS spectrum of Loganin in urine.

Based on the chemical characterization of XEASD, the MS/MS fragmentation patterns and retention time were used to analyze the components in plasma, urine, feces, and bile. **P53** Loganin was selected as an example, and its XIC in XEASD (Figure 4A) and multiple XICs of 8 bio-samples (Figure 4B) were examined. A peak at 9.35 min was clearly observed, which only appeared in the administered urine of bio-samples and responded at the same retention time as the extracts. Of great significance, the MS/MS spectra (*m/z* of 435, 227, and 127) of Loganin in XEASD (Figure 4C) and bio-samples (Figure 4D) were found to be comparable.

Based on the previous results, a total of 54 prototypes were detected from plasma, urine, feces, or bile samples. Among them, 2 compounds were detected in plasma, 27 in urine, 44 in feces, and 6 in bile. These compounds in feces may not be absorbed into the bloodstream, which could be still helpful in modulating the fecal microbiota. Elaborate process distribution of the prototypes is shown in Table 2.

**TABLE 2:** Distribution of prototype compounds *in vivo*.

NO.	Name	Bile	Brain	Feces	Heart	Kidney	Liver	Lung	Plasma	Spleen	Urine
P20	N-Methyltyramine	-	-	√	-	-	-	-	-	-	√
P22	Hordenine	-	-	√	-	-	-	-	-	-	-
P32	3,4-dihydroxybenzaldehyde	-	-	√	-	-	-	-	-	-	√
P35	Morroniside	-	-	-	-	-	-	-	-	-	√
P36	Polygenin C	-	-	√	-	-	-	-	-	-	-
P39	Polygenin C isomer	-	-	√	-	-	-	-	-	-	-
P40	2-benzyl-2,3-dihydroxybutanedioic acid	-	-	-	-	-	-	-	-	-	√
P41	Sibiricose A1	-	-	√	-	-	-	-	-	-	-
P44	Cnidioside A	-	-	√	-	-	-	-	-	-	-
P45	Esculetin	-	-	√	-	-	-	-	-	-	√
P46	Polygalatenosides A	-	-	√	-	-	-	-	-	-	-
P47	Sibiricose A1 isomer	-	-	√	-	-	-	-	-	-	-
P51	Puerarin	-	-	√	-	-	-	-	-	-	-
P53	Loganin	-	-	-	-	-	-	-	-	-	√
P56	Sweroside	-	-	-	-	-	-	-	-	-	√
P68	Sibiricaxanthone B	-	-	√	-	-	-	-	-	-	-
P70	Glomeratose A	-	-	√	-	-	-	-	-	-	-
P73	Polygalaxanthone III	-	-	√	-	-	-	-	-	-	-
P74	Irisxanthone	-	-	√	-	-	-	-	-	-	-
P77	Oxyphyllenone B	-	-	√	-	-	-	-	-	-	√
P81	Liquiritin apioside	-	-	√	-	-	-	-	-	-	√
P83	Umbelliferone	-	-	√	-	-	-	-	-	-	√
P84	Limonin glucoside	-	-	√	-	-	-	-	-	-	-
P85	Eriocitrin	-	-	√	-	-	-	-	-	-	-
P87	Liquiritin	-	-	-	-	-	-	-	-	-	√
P92	Sinapic acid	-	-	√	-	-	-	-	-	-	√
P94	2,4,5-trimethoxybenzoic acid	-	-	√	-	-	-	-	√	-	√
P96	Marmesinin	√	-	√	-	-	-	-	-	-	√
P97	Naringin	-	-	√	-	-	-	-	-	-	-
P101	Rhoifolin	√	-	√	-	-	-	-	-	-	-
P103	Narirutin	√	-	√	-	-	-	-	-	-	-
P107	Hesperidin	-	-	√	-	-	-	-	-	-	-
P111	Neohesperidin	-	-	√	-	-	-	-	-	-	-
P113	Salicylic acid	-	-	√	-	-	-	-	√	-	√
P116	Tenuifoliside A	-	-	√	-	-	-	-	-	-	-
P117	Nomilinic acid glycoside	-	-	√	-	-	-	-	-	-	-
P124	Teuhetenone	-	-	-	-	-	-	-	-	-	√
P125	5-Isopentenyloxy-7-methoxycoumarins	-	-	√	-	-	-	-	-	-	-
P129	Tenuifoliose J	-	-	√	-	-	-	-	-	-	-
P131	Marmesin	-	-	√	-	-	-	-	-	-	√
P135	Oroxylin A Glucoronide	√	√	-	-	-	-	-	-	-	√
P136	Liquiritigenin	-	-	√	-	-	-	-	-	-	√
P142	3, 4, 5-trimethoxy-trans-cinnamic acid	-	-	√	-	-	-	-	-	-	-
P146	Calycosin	-	-	√	-	-	-	-	-	-	√
P147	Citrusin III	√	-	√	-	-	-	-	-	-	-
P151	Melitidin	√	-	√	-	-	-	-	-	-	-
P157	Naringenin	-	-	√	-	-	-	-	-	-	√
P162	Homoeriodictyol	-	-	√	-	-	-	-	-	-	√
P168	Monohydroxy-tetramethoxyflavone	-	-	-	-	-	-	-	-	-	√
P173	Isoliquiritigenin	-	-	√	-	-	-	-	-	-	√
P174	Glycyrrhizic acid	-	-	√	-	-	-	-	-	-	-
P175	7-hydroxy-5,6,8,3′,4′-pentamethoxyflavone	-	-	-	-	-	-	-	-	-	√
P179	Formononetin	-	-	√	-	-	-	-	-	-	√
P186	Oxyphyllenone H	-	-	-	-	-	-	-	-	-	√
Total		6	1	44	0	0	0	0	2	0	27

**FIGURE 5:**
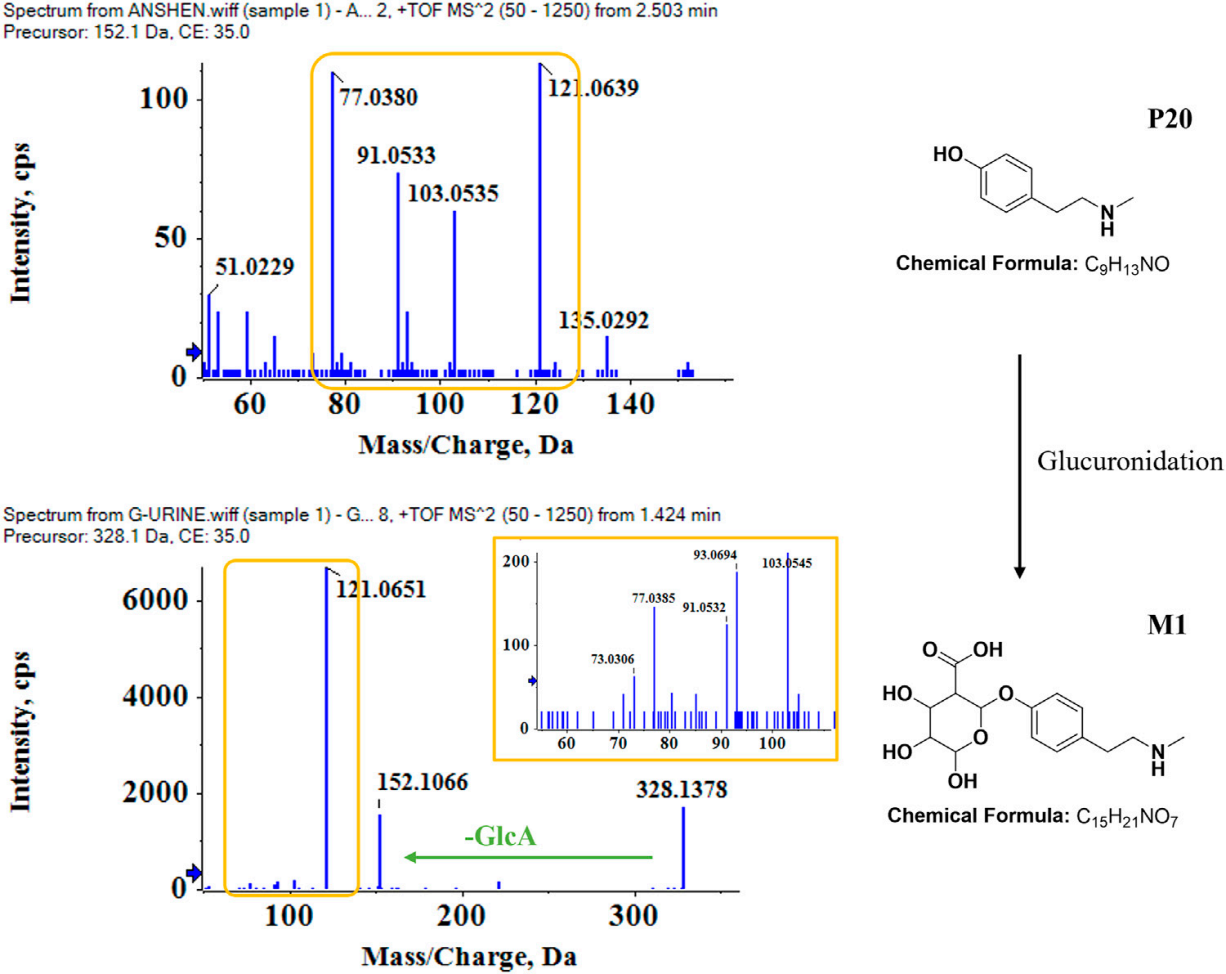
Identification of metabolites in bio-samples. Metabolite identification and matching process based on the similarity of the cleavage pattern and profile of the P20 N-Methyltyramine-M1 secondary mass spectrum.

**FIGURE 6:**
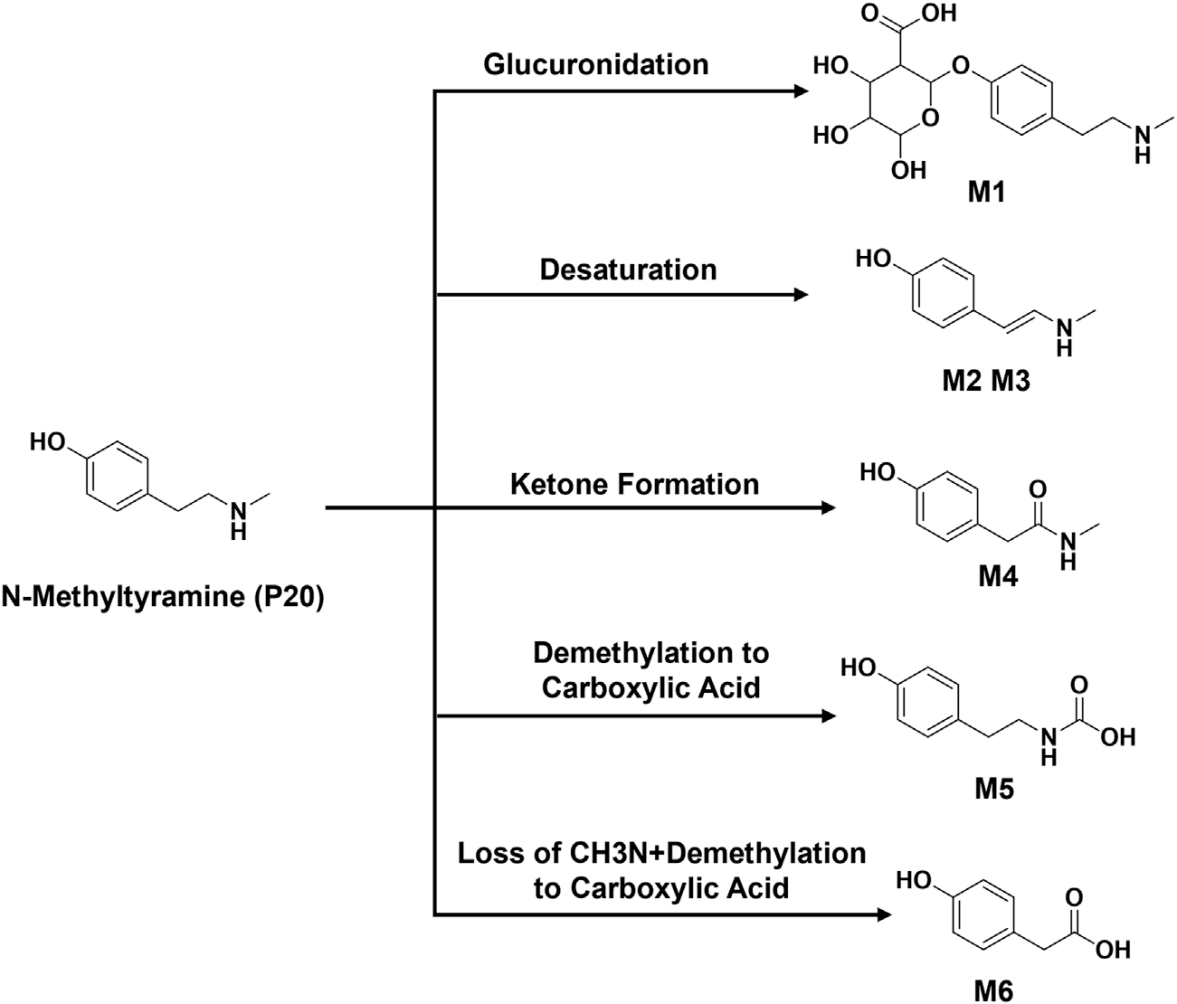
Structural association of N-Methyltyramine with metabolites.

The metabolic patterns of phase I and phase II were used as the basis for the similarity of secondary mass spectrometry profiles to achieve rapid screening of metabolite libraries from the matrix that are distinct from the prototype components. This automatic matching the prototype components assist in the identification and annotation of metabolites, as illustrated in Figure 5. For example, the mass deviation between **P20** and **M1** were Δm = 176.0318, which is consistent with the biotransformation pathway “glucuronidation”. Comparing the secondary mass spectra of **P20** and **M1**, it was possible to spot the distinctive fragments produced when the GlcA group was taken out of **M1**. There is a high similarity between the two secondary profiles, including *m/z* 121, 103, 93, 91, 77, etc. Therefore, **M1** can be matched as one of the metabolites of **P20**. Following this principle, a total of six metabolites were matched to **P20**, and their structural association diagrams are presented in Figure 6.

**FIGURE 7:**
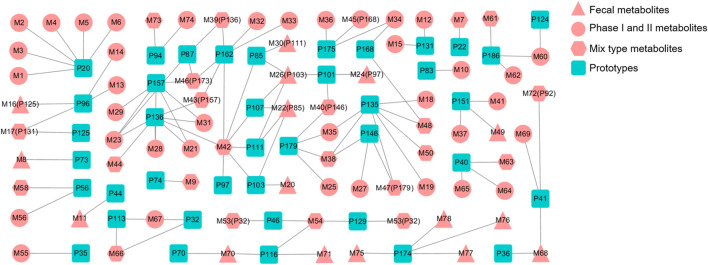
Correlation between prototype and metabolites.

In this regard, further metabolite analysis was performed on the 54 prototype compounds mentioned earlier, of which 41 prototypes could be matched to metabolites, resulting in a total of 78 matched metabolites. Among them, four were detected in plasma, fifty-eight in urine, forty in feces and twenty-three in bile. The detailed distribution of metabolites has been shown in Table 3. Figure 7 shows the association network between the prototypes and the related metabolites. Metabolites detected in feces are assumed to be metabolized by intestinal flora, while those metabolized by the liver may be detected in bile, plasma, and urine. Detailed biotransformation and annotation of prototype and metabolite components are presented in Supplementary Table S3.

**TABLE 3:** Metabolite distribution *in vivo*.

Metabolites	Bile	Brain	Feces	Heart	Kidney	Liver	Lung	Plasma	Spleen	Urine
M1	-	-	-	-	-	-	-	-	-	√
M2	-	-	-	-	-	-	-	-	-	√
M3	-	-	-	-	-	-	-	-	-	√
M4	-	-	-	-	-	-	-	-	-	√
M5	-	-	-	-	-	-	-	-	-	√
M6	-	-	-	-	-	-	-	-	-	√
M7	-	-	√	-	-	-	-	-	-	√
M8	-	-	√	√	-	-	-	-	-	-
M9	-	-	√	-	-	-	-	-	-	√
M10	-	-	-	-	-	-	-	-	-	√
M11	-	-	√	-	-	-	-	-	-	-
M12	-	-	-	-	-	-	-	-	-	√
M13	√	-	-	-	-	-	-	-	-	-
M14	√	-	-	-	-	-	-	-	-	-
M15	-	-	-	-	-	-	-	-	-	√
M16(P125)	-	-	√	-	-	-	-	-	-	-
M17(P131)	-	-	√	-	-	-	-	-	-	√
M18	√	-	-	-	-	-	-	-	-	-
M19	√	-	-	-	-	-	-	-	-	√
M20	√	-	√	-	-	-	-	-	-	-
M21	√	-	-	-	-	-	-	-	-	√
M22(P85)	-	-	√	-	-	-	-	-	-	-
M23	-	-	-	-	-	-	-	-	-	√
M24(P97)	-	-	√	-	-	-	-	-	-	-
M25	-	-	-	-	-	-	-	-	-	√
M26(P103)	√	-	√	-	-	-	-	-	-	-
M27	√	-	-	-	-	-	-	-	-	√
M28	-	-	-	-	-	-	-	-	-	√
M29	-	-	-	-	-	-	-	-	-	√
M30(P111)	-	-	√	-	-	-	-	-	-	-
M31	√	-	-	-	-	-	-	-	-	√
M32	√	-	-	-	-	-	-	-	-	√
M33	√	-	-	-	-	-	-	-	-	√
M34	√	-	-	-	-	-	-	-	-	√
M35	√	-	-	-	-	-	-	-	-	√
M36	√	-	-	-	-	-	-	-	-	√
M37	√	-	-	-	-	-	-	-	-	-
M38	-	-	√	-	-	-	-	-	-	√
M39(P136)	-	-	√	-	-	-	-	-	-	√
M40(P146)	-	-	√	-	-	-	-	-	-	√
M41	√	-	-	-	-	-	-	-	-	√
M42	-	-	√	-	-	-	-	-	-	√
M43(P157)	-	-	√	-	-	-	-	-	-	√
M44	-	-	√	-	-	-	-	-	-	√
M45(P168)	-	-	-	-	-	-	-	-	-	√
M46(P173)	-	-	√	-	-	-	-	-	-	√
M47(P179)	-	-	√	-	-	-	-	-	-	√
M48	-	-	√	-	-	-	-	-	-	√
M49	-	-	√	-	-	-	-	-	-	-
M50	-	-	√	-	-	-	-	-	-	√
M51	-	-	√	-	-	-	-	-	-	√
M52	-	-	√	-	-	-	-	-	-	√
M53(P32)	-	-	√	-	-	-	-	-	-	√
M54	√	-	√	-	√	-	-	√	-	√
M55	√	-	-	-	-	-	-	-	-	√
M56	√	-	-	-	-	-	-	-	-	√
M57	√	-	-	-	-	-	-	-	-	√
M58	-	-	√	-	-	-	-	-	-	√
M59	-	-	√	-	-	-	-	√	-	√
M60	√	-	-	-	-	-	-	-	-	√
M61	-	-	√	-	-	-	-	√	-	√
M62	-	-	-	-	-	-	-	√	-	√
M63	-	-	√	-	-	-	-	-	-	√
M64	-	-	-	-	-	-	-	-	-	√
M65	-	-	-	-	-	-	-	-	-	√
M66	-	-	√	-	-	-	-	-	-	√
M67	-	-	-	-	-	-	-	-	-	√
M68	√	-	√	-	-	-	-	-	-	-
M69	-	-	-	-	-	-	-	-	-	√
M70	-	-	√	-	-	-	-	-	-	-
M71	-	-	√	-	-	-	-	-	-	-
M72(P92)	-	-	√	-	-	-	-	-	-	√
M73	-	-	√	-	-	-	-	-	-	√
M74	-	-	-	-	-	-	-	-	-	√
M75	-	-	√	-	-	-	-	-	-	-
M76	√	-	√	-	-	√	-	-	-	-
M77	-	-	√	-	-	-	-	-	-	-
M78	-	-	√	-	-	-	-	-	-	-
Total	23	0	40	1	1	1	0	4	0	58

**FIGURE 8:**
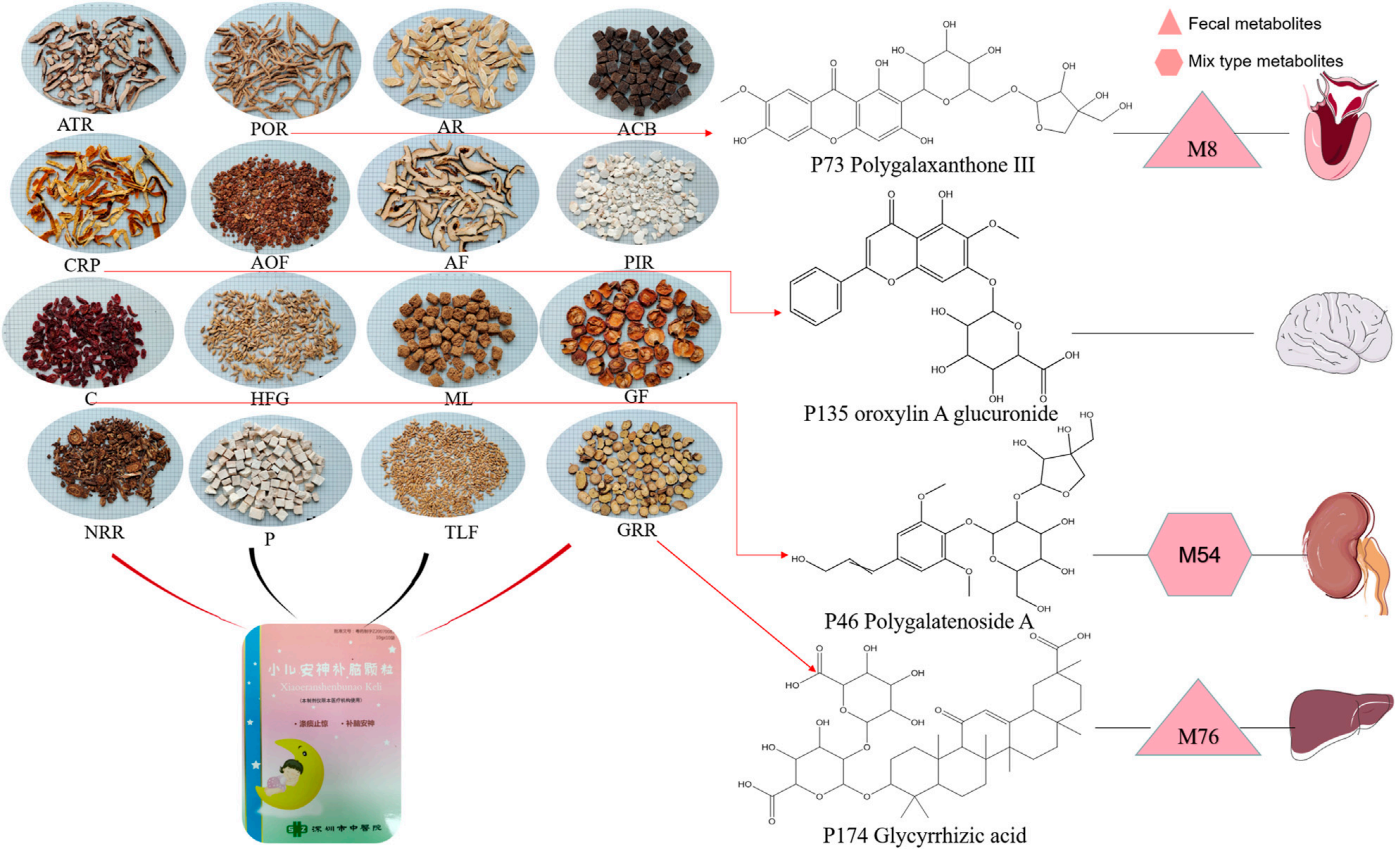
Herbal ingredients form XEASD and major compounds with potential therapeutic effects identified in various organs.

The analysis of tissue distribution of compounds mentioned above showed that a limited number of compounds were distributed in tissues, and none were detected in lung and spleen tissues. Specifically, **P135** Oroxylin A glucuronide was the only prototype detected in the brain tissue, and **P73** Polygalaxanthone III was metabolized into **M8**, which was identified in the heart tissue. Moreover, **P46** Polygalatenoside A was metabolized into **M54**, which was detected in the kidney tissue, while **P174** Glycyrrhizic acid was metabolized into **M76** and identified in the liver tissue (Figure 8). These ingredients are believed to be the underlying chemical basis for the therapeutic effects of XEASD.

### 3.4 Oroxylin A glucuronide operates positive effect in inhibiting oxidative stress *in vitro*

**FIGURE 9:**
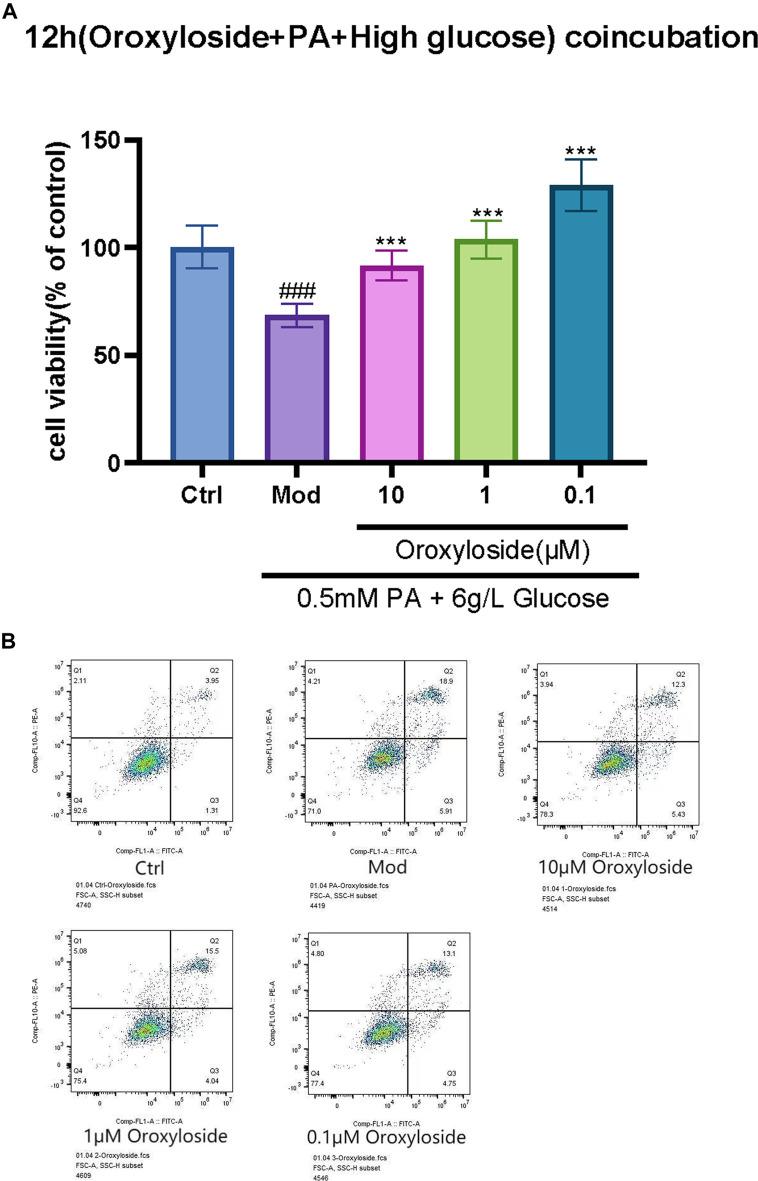
Effect of Oroxylin A glucuronide on the proliferation and apoptosis in H9C2 cells. **(A)** H9C2 cells were treated with Oroxylin A glucuronide (1, 10, and 100 µm) for 12 h and cell viability was measured by MTT assay. **(B)** The apoptosis-inducing effect of Oroxylin A glucuronide on H9C2 cells was tested by Annexin-V FITC/PI double-staining assay. Each experiment was done independently at least three times and all the data is quantified as the mean ± SEM. ** *p*-value < 0.01 and *** *p*-value < 0.001 compared with the model group and **###** *p*-value < 0.001 compared with the control group.

The results of cell viability assay (Figure 9A) and Annexin-V FITC/PI staining (Figure 9B) showed that 12-hr treatment of Oroxylin A glucuronide (Figure 8) at indicated concentrations (1, 10, and 100 µm) led to an enhanced effect on cell viability, and the apoptotic proportion was decreasing by Oroxylin A glucuronide in a dose-dependent manner, indicating that Oroxylin A glucuronide inhibited oxidative stress in cardiomyocytes with high glucose stimulation.

## 4 Discussion

These results of our study indicate that XEASD contains flavonoids, phenylpropanoids, triterpenoids and amino acids as its major chemical constituents.

Our findings build on prior work (Hou-ming. et al., 2018) by highlighting the importance of chemical characterization and metabolic profiling of XEASD. While GAO et al. used a single selective condition (HPLC-MS) to detect eight components (liquiritin, calycosin-7-O-β-Dglucoside, ammonium glycyrrhizinate, naringin, 3,6′-disinapoyl sucrose, hesperidin, neohesperidin, and astragaloside Ⅳ) in XEASD for quality control, our study analysed 54 prototypes of XEASD in various bodily fluids, including plasma, urine, feces, and bile. A total of 78 metabolites were discovered after 41 of these prototypes could be matched to existing metabolites. Overall, our study provides a more comprehensive understanding of the chemical composition and metabolic fate of XEASD. We were able to learn how XEASD is metabolized in the body by examining a variety of prototypes *in vivo*, which has implications for future studies on theeffectiveness and safety of XEASD.

**P83** Umbelliferone (Zagaja et al., 2022) and **P111** Neohesperidin (Chakraborty et al., 2021) have been reported to exhibit neuroprotective effects. **P136** Liquiritigenin (Liu et al., 2022), **P116** Tenuifoliside A (Dong et al., 2014), **P73** Polygalaxanthone III (Du et al., 2022), **P53** Loganin (Pan et al., 2021) and **P151** Natsudaidain 3-(4-O-3-Hydroxy-3-Methylglutaroylglucoside) (Okuyama et al., 2017) have been shown to exert their effects through various mechanisms, including changes in neurotransmitter levels, increased cAMP levels and amelioration of locomotor hyperactivity through activation of the ERK/CREB signaling pathway. **P107** Hesperidin (Hajialyani et al., 2019), **P179** Formononetin (Fang et al., 2020; Tian et al., 2022), **P35** Morroniside(Xu et al., 2017), **P162** Homoeriodictyol (Guo et al., 2022) and **P22** Hordenine (Rom et al., 2022; Su et al., 2022) could exert neuroprotection by reducing neuroinflammation and oxidative stress. **P162** Homoeriodictyol and **P22** Hordenine have also been found to cross the blood-brain barrier directly. **P103** Narirutin (Mitra et al., 2022; Yang et al., 2022), **P157** Naringenin (Goyal et al., 2022), **P87** Liquiritin (Qin et al., 2022), **P101** Rhoifolin (Brinza et al., 2020; Mai et al., 2022), **P146** Calycosin (Deng et al., 2021), **P97** Naringin (Ahmed et al., 2019; Zhou et al., 2019), **P56** Sweroside (Yang et al., 2020; Brinza et al., 2022) and **P174** Glycyrrhizic acid(Cao et al., 2020) have been demonstrated to possess therapeutic effects in a variety of neurological disorders, such as antidepressants, as well as protecting the liver. Furthermore, **P85** Eriocitrin (Meng et al., 2022) and **P168** Monohydroxy-tetramethoxyflavone (Zheng et al., 2020), have shown beneficial effects on microbiota and bacterial diversity, thereby improving wasting muscle atrophy or ameliorating splenomegaly-related diseases. In contrast, to recent work that focused solely on *in vitro* chemical composition analysis and network pharmacology analysis of a TCM-derived product (Jingxin Zhidong Formula) for TD treatment (Tian et al., 2022), our study analyzed *in vivo* blood components and tissue distribution, as well as correlated *in vitro* chemical composition prototypes and metabolized metabolites. These compounds may have potential therapeutic applications for various conditions and represent important quality control markers for XEASD.

These findings shed new light on the mechanisms underlying neurodegeneration in TD diseases. For instance, **P135** Oroxylin A glucuronide was detected in brain tissue, and *in vitro* experiments have demonstrated that it can effectively inhibit high glucose-stimulated cardiomyocytes, which was tightly associated with oxidative stress (Chen et al., 2023), while *in vitro* and *in vivo* studies have shown that the main mechanism of XEASD for TD is closely related to the inhibition of oxidative stress.

However, identification of active compounds is a critical first step in the mechanistic analysis of how herbal macrobiotics affect various aspects of body functions, once these key compounds have been identified, further extensive work is required. For example, it is important to determine whether prototypical compounds involved in metabolic reactions are closely linked to the progression of specific diseases. Another crucial objective is the identification of target molecules affected by the active components and the key signaling pathways involved in their biological functions. The present study exemplifies the identification and characterization of cleavage patterns of potentially key compounds or metabolites in the context of specific diseases and herbal formulas, providing valuable insights for the development of targeted therapies.

## 5 Conclusion

The potentially major compounds identified in XEASD were flavonoids, phenylpropanoids, triterpenoids, and amino acids. The chemical basis underlying the beneficial effects of XEASD against TD may be attributed to active compounds such as **P46** Polygalatenoside A, **P73** Polygalaxanthone III, **P135** Oroxylin A Glucuronide, **P174** Glycyrrhizic acid as well as the metabolites of liver metabolisms and fecal metabolites. In conclusion, this study highlights the need for further pharmacokinetic and pharmacological evaluation of XEASD.

## Figures and Tables

**Table:** 

**TD**	Tic disorder
**TS**	Tourette syndrome
**OCD**	Obsessive-compulsive disorder
**ADHD**	Attention-deficit/hyperactivity disorder
**cAMP**	cyclic adenosine monophosphate
**CREB**	cAMP responsive element-binding protein
**BSA**	Bovine serum albumin
**D2R**	Dopamine receptor type 2
**MDF**	Mass defect filtration
**ATR**	Acori tatarinowii Rhizoma
**POR**	Polygalae Radix
**AR**	Astragali Radix
**ACB**	Arisaema Cum Bile
**CRP**	Citri reticulatae pericarpium
**AOF**	Alpiniae oxyphyllae Fructus
**AF**	Aurantii Fructus
**PIR**	Pinelliae Rhizoma
**NRR**	Notopterygii Rhizoma et Radix
**P**	Poria
**TLF**	Tritici Levis Fructus
**GRR**	Glycyrrhizae Radix et Rhizoma
**C**	Corni Fructus
**HFG**	Hordei Fructus Germinatus
**ML**	Massa Medicata Fermentata
**GF**	Crataegi Fructus
**Con**	Control
**HPLC**	High Performance Liquid Chromatography
**XEASD**	Xiao-Er-An-Shen Decoction
**TCM**	Traditional Chinese medicine
**TCMSP**	Traditional Chinese Medicine Systems Pharmacology
**DMSO**	Dimethyl sulfoxide
**FA**	Formic acid
**Glc**	Glucose
**GlcA**	Glucuronic acid
**NS**	Normal saline
**UPLC**	Ultra Performance Liquid Chromatography
**SPE**	Solid-phase extraction
**OD**	Optical density
**CES**	Collision energy spread
**XIC**	Extracted ion chromatogram
**BPC**	Base peak chromatogram
**TIC**	Total ion chromatogram

## Data Availability

The original contributions presented in the study are included in the article/Supplementary Material, further inquiries can be directed to the corresponding authors.

## Supplementary material

SUPPLEMENTARY FIGURE S1 Decoction samples of 16 Chinese herbal medicines in XEASD. **(A)** Acori tatarinowii Rhizoma; **(B)** Polygalae Radix; **(C)** Astragali Radix; **(D)** Arisaema Cum Bile; **(E)** Citri reticulatae pericarpium; **(F)** Alpiniae oxyphyllae Fructus; **(G)** Aurantii Fructus; **(H)** Pinelliae Rhizoma; **(I)** Notopterygii Rhizoma et Radix; **(J)** Poria; **(K)** Fructus Tritici Levis; **(L)** Glycyrrhizae Radix et Rhizoma; **(M)** Cornus; **(N)** Hordei Fructus Germinatus; **(O)** Massa Medicata Fermentata; **(P)** Crataegi Fructus.

SUPPLEMENTARY FIGURE S2 Base peak chromatogram (BPC) of plasma.

SUPPLEMENTARY FIGURE S3 Base peak chromatogram (BPC) of urine.

SUPPLEMENTARY FIGURE S4 Base peak chromatogram (BPC) of feces.

SUPPLEMENTARY FIGURE S5 Base peak chromatogram (BPC) of bile.
