# Supplementary figures and images for: Chemical characterization and metabolic profiling of Xiao-Er-An-Shen Decoction by UPLC-QTOF/MS
Source: Front Pharmacol. 2023 Nov 2;14:1219866. doi: 10.3389/fphar.2023.1219866 (PMC10652787; doi:10.3389/fphar.2023.1219866)

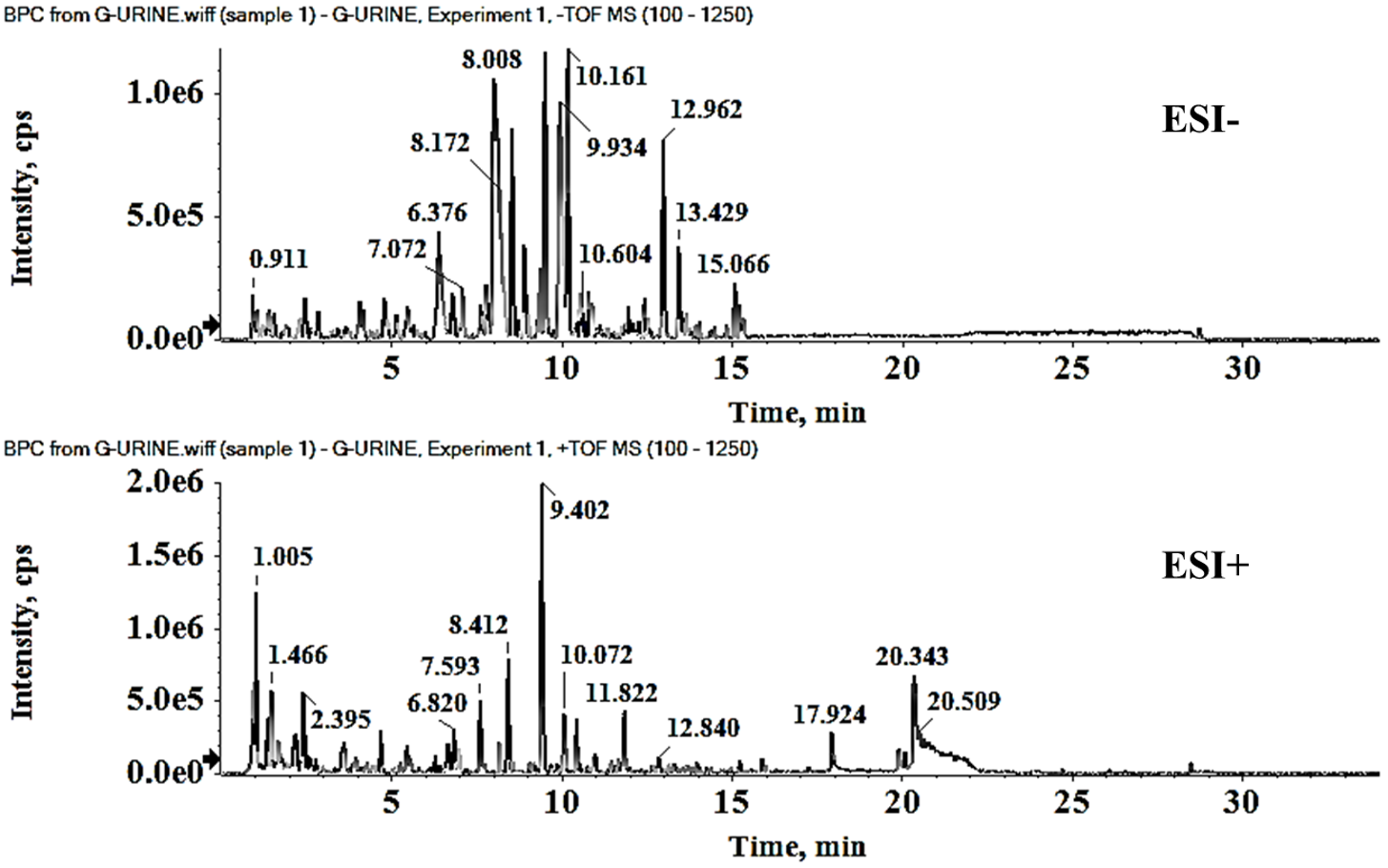

Supplement: Supplementary file 3 [file Image3.TIF]

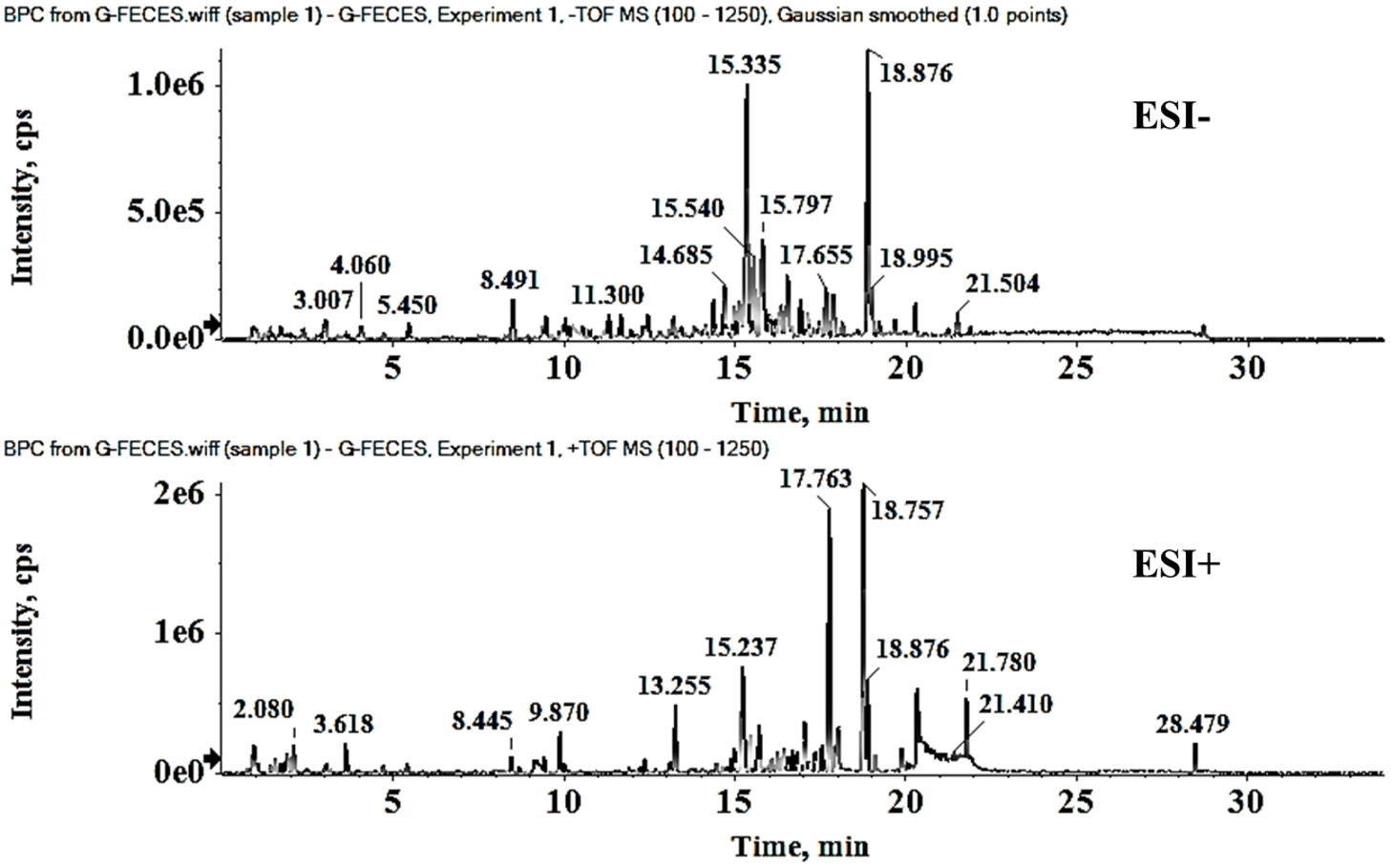

Supplement: Supplementary file 4 [file Image4.TIF]

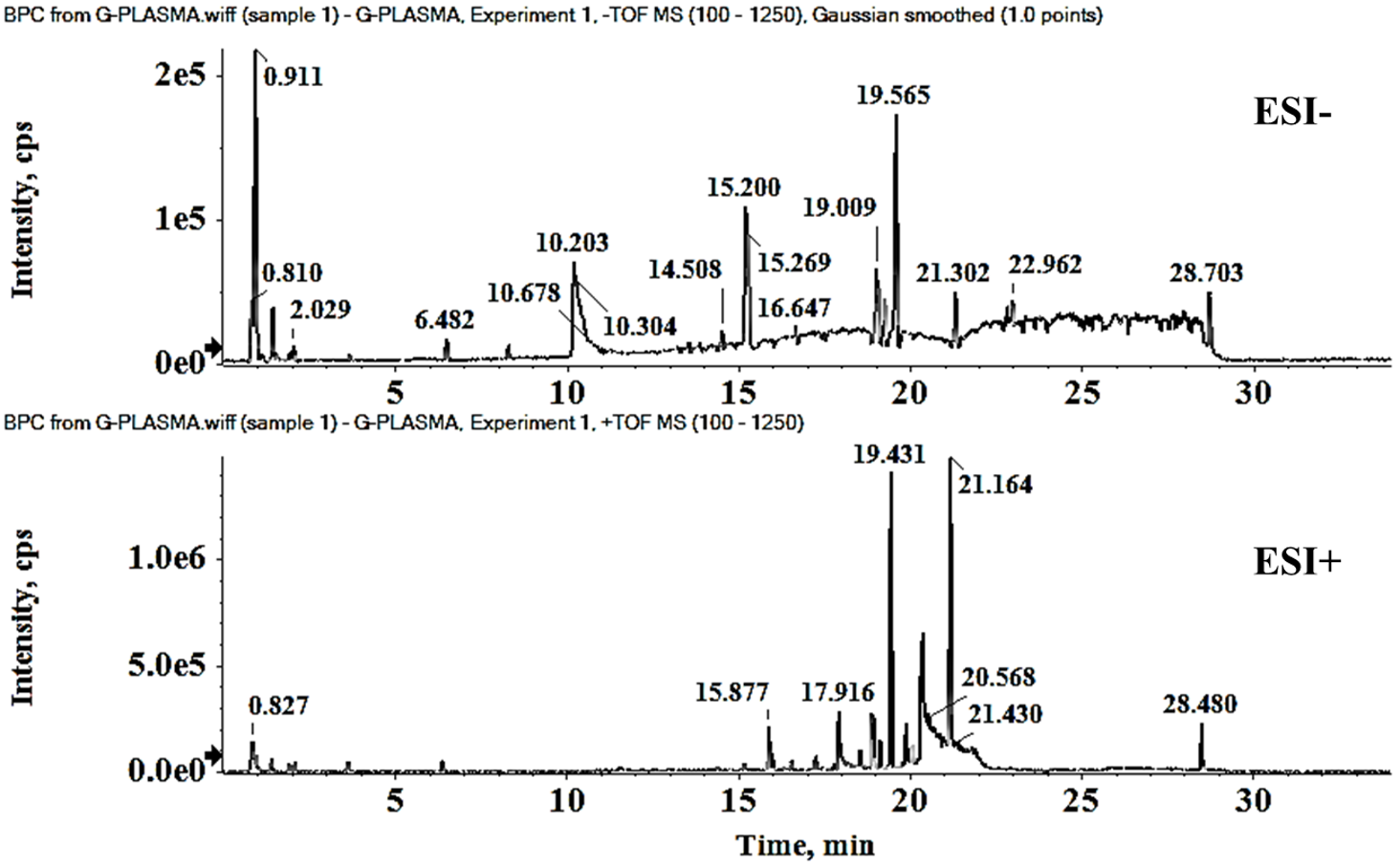

Supplement: Supplementary file 5 [file Image2.TIF]

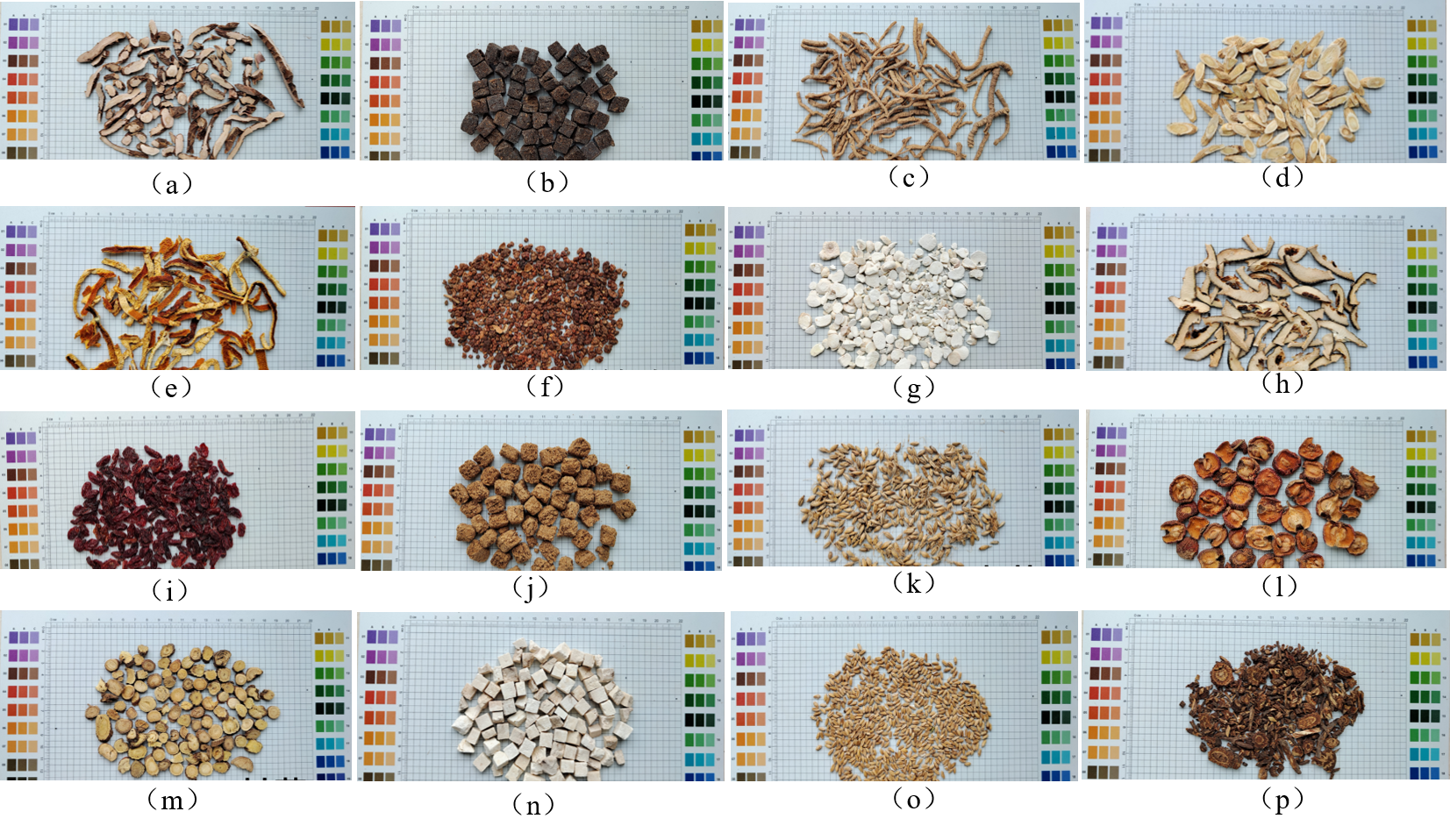

Supplement: Supplementary file 6 [file Image1.TIF]

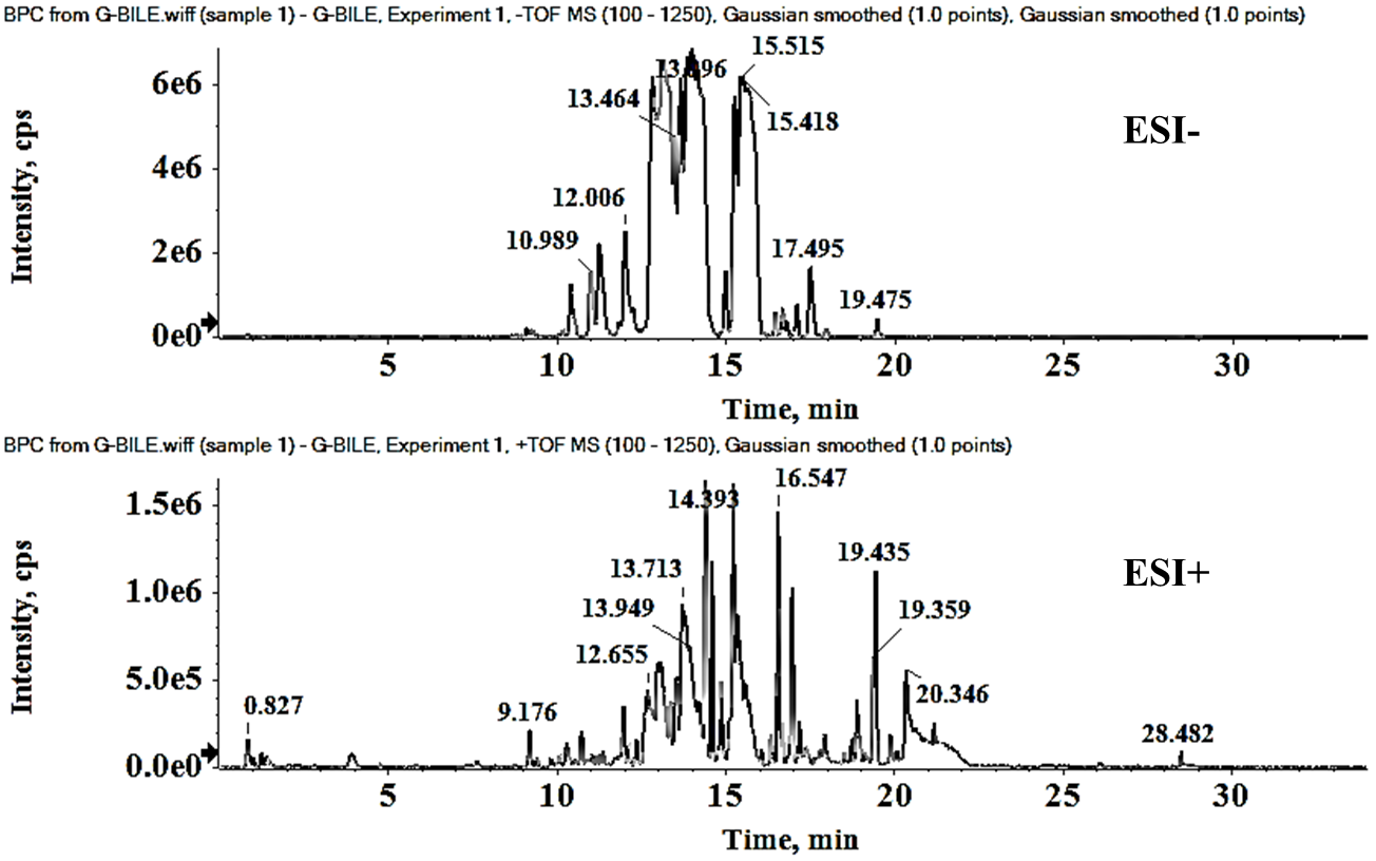

Supplement: Supplementary file 9 [file Image5.TIF]
