# Supplementary material for: Chemical characterization and metabolic profiling of Xiao-Er-An-Shen Decoction by UPLC-QTOF/MS
Source: Front Pharmacol. 2023 Nov 2;14:1219866. doi: 10.3389/fphar.2023.1219866 (PMC10652787; doi:10.3389/fphar.2023.1219866)

UPLC-MS/MS spectra for fragmentation pattern of identified molecules.

Phenylpropanoids: P38 Chlorogenic acid


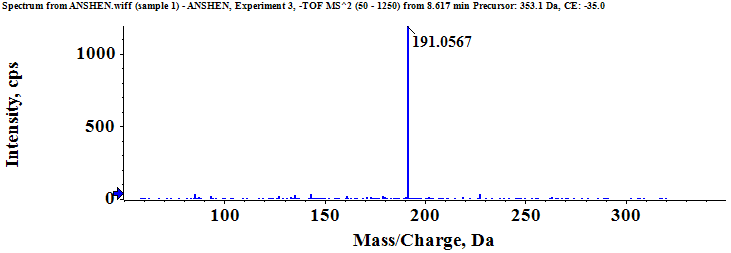


Phenylpropanoids glycoside: P41 Sibiricose A1


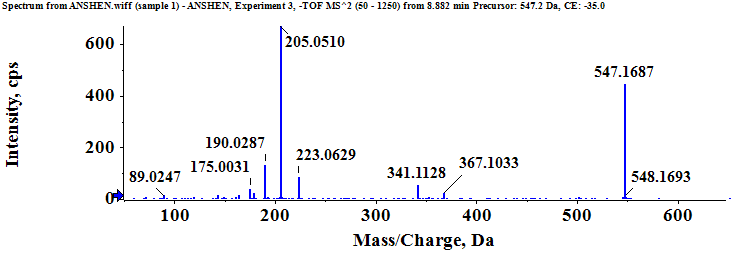


Anthranones: P68 Sibiricaxanthone B


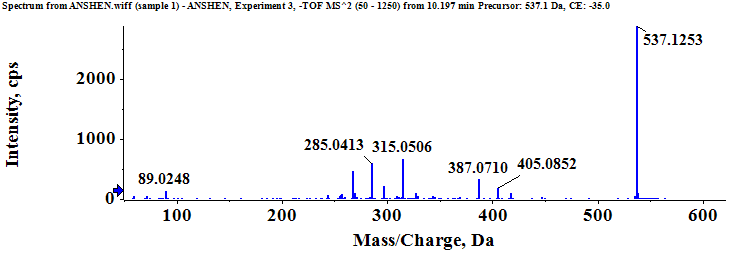


Alkaloids: P147 Citrusin III


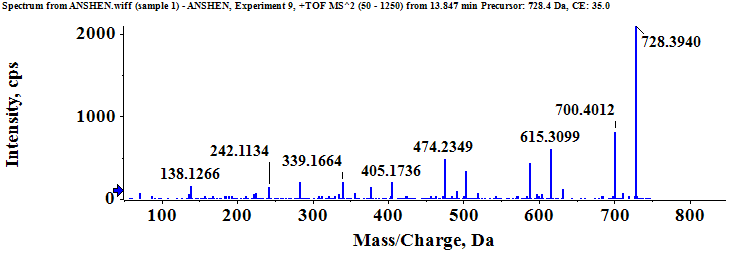


Flavonoids: P57 Vicenin-2 (C-glycoside)


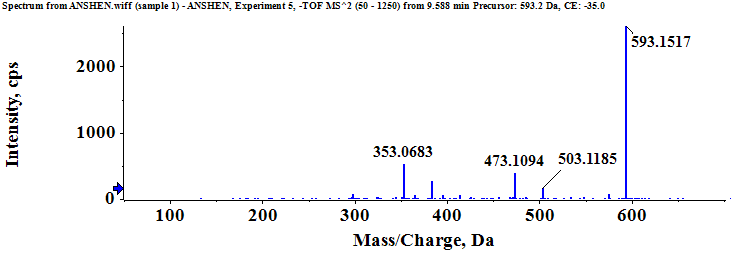


Limonoids: P189 Limonin


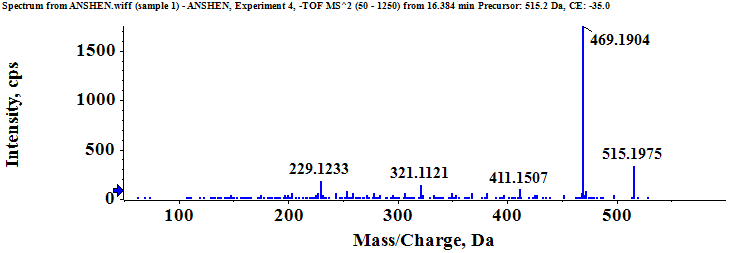


Ketones: P48 Oxyphyllenone A


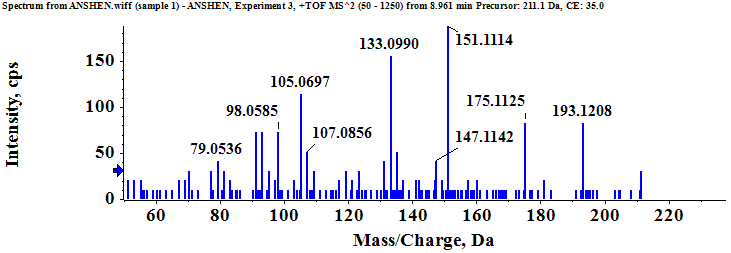


Coumarins: P72 Psoralen


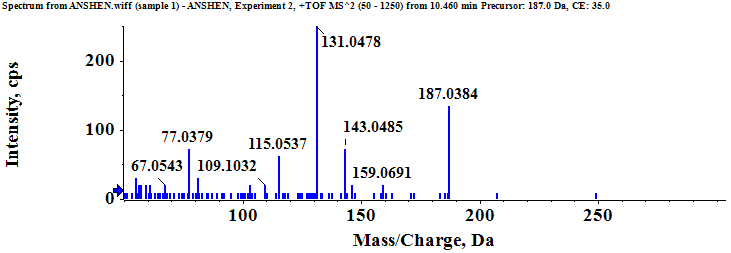


Flavonoids: P87 Liquiritin (O-glycoside)


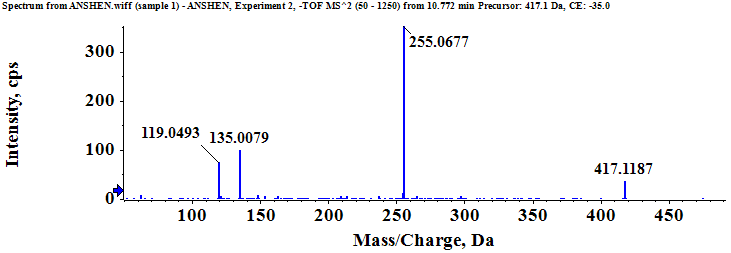


Triterpenoid saponins: P145 Uralsaponin F


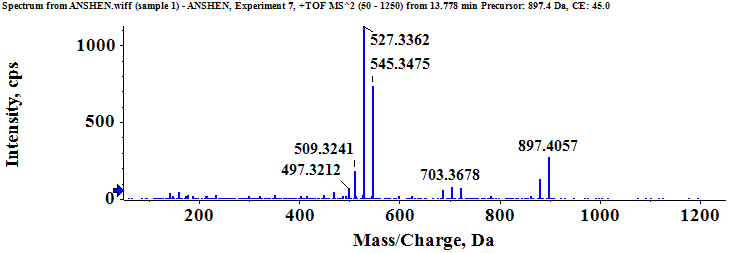


Triterpenoid acids: P172 Pinicolic acid E


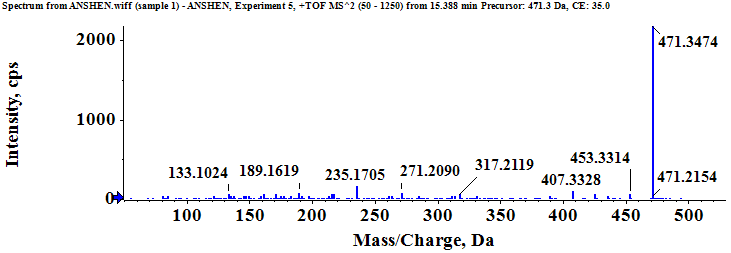


Iridoid glycosides: P53 Loganin


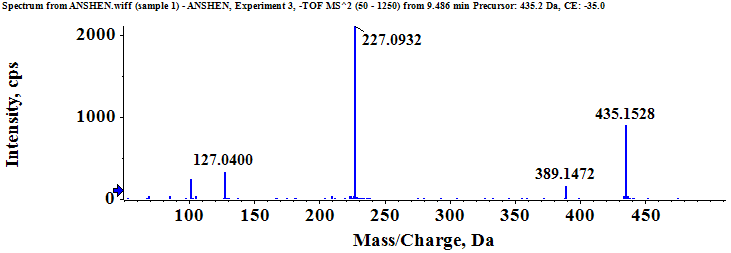


Organic acids: P166 Pinellic Acid


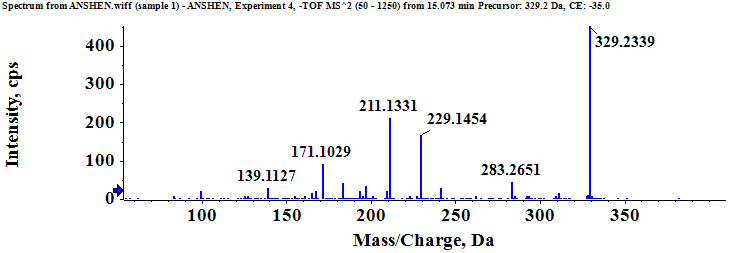

Supplement: Supplementary file 8 [file Table4.DOCX]
